# Berberine induces PD-L1 degradation via the autophagy-lysosome pathway through the PI3K-Akt pathway and enhances immunogenic cell death in triple-negative breast cancer

**DOI:** 10.3389/fimmu.2026.1797840

**Published:** 2026-08-07

**Authors:** Yuqin Wei, Qiudan Huang, Xingwen Wei, Ruilan Mo, Wei Zhao

**Affiliations:** 1 Department of Comprehensive Cancer Center (Ward 2), The Second Affiliated Hospital of Guangxi Medical University, Nanning, China; 2 Department of Radiotherapy, Guangxi Medical University Cancer Hospital, Nanning, China; 3 Department of Respiratory Medicine, Wuming Hospital of Guangxi Medical University, Nanning, China

**Keywords:** Berberine, autophagy-lysosome, PD-L1, PI3K-Akt, triple-negative breast cancer

## Abstract

**Objective:**

This study aimed to investigate the regulatory effect and molecular mechanism of Berberine (BBR) on programmed death-ligand 1 (PD-L1) expression in Triple-Negative Breast Cancer (TNBC) cells.

**Methods:**

*In vitro* cellular experiments (using MDA-MB-231 and BT-549 cell lines) and mouse models (Balb/c-nu and Balb/c mice) were performed. Combined with techniques including Western blot and immunofluorescence assay, the effect of BBR on PD-L1 expression was evaluated.

**Results:**

BBR reduced PD-L1 protein expression in TNBC cells in a time- and dose-dependent manner, decreased the membrane localization of PD-L1, and inhibited interferon-γ (IFN-γ)-induced PD-L1 upregulation. Mechanistically, BBR activated autophagy by inhibiting the PI3K-Akt pathway, leading to PD-L1 degradation primarily mediated by the autophagy-lysosome pathway, with minimal contribution from the proteasome under the conditions tested. Cycloheximide (CHX) pulse-chase assay confirmed the degradation kinetics of PD-L1. Experiments evaluating lysosomal function excluded the influence of abnormal lysosomal function, demonstrating that this degradation was independent of the ubiquitin-proteasome system and did not affect lysosomal function. Furthermore, BBR increased the expression and release of immunogenic cell death (ICD)-associated markers in TNBC cells, including calreticulin (CRT) exposure and high-mobility group box 1 (HMGB1) release. *In vivo* experiments showed that BBR treatment did not significantly reduce tumor volume or weight in Balb/c-nu mice, but effectively decreased tumor volume and weight in the 4T1 Balb/c mouse model without obvious systemic toxicity. In the tumor tissues of 4T1 Balb/c mice, the proportion of CD8^+^CD3^+^ and CD69^+^CD8^+^ activated T cells was significantly increased, the expression level of granzyme B in CD8^+^ T cells was notably elevated, and the number of regulatory T cells (Tregs, CD4^+^CD25^+^Foxp3^+^) was significantly reduced.

**Conclusion:**

BBR can regulate PD-L1 degradation and induce ICD through the PI3K-Akt-autophagy pathway. Its mode of immune checkpoint degradation and immunogenic activation provides experimental evidence for the immunotherapy of TNBC.

## 1 Introduction

Triple-negative breast cancer (TNBC) is an aggressive subtype lacking estrogen receptor, progesterone receptor, and human epidermal growth factor receptor 2 expression. Due to the absence of effective targeted therapies, TNBC patients exhibit unfavorable prognosis and low survival rates (1). Immune checkpoint blockade (ICB) has revolutionized cancer treatment, yet its clinical response in TNBC remains modest. The major obstacles include low tumor immunogenicity and an immunosuppressive tumor microenvironment (TME) that impairs the function of tumor-infiltrating lymphocytes, thereby limiting the efficacy of immunotherapy (2, 3). Therefore, developing effective strategies to restore immune surveillance and enhance immunotherapy responsiveness in TNBC is urgently needed.

Programmed death-ligand 1 (PD-L1) is a pivotal immune checkpoint that is highly expressed in TNBC and drives immune escape by suppressing cytotoxic T cell activity. PD-L1 expression is tightly regulated at multiple levels, and post-translational control critically determines its protein stability and membrane localization (4, 5). Accumulating evidence indicates that the autophagy-lysosome pathway, independent of the ubiquitin-proteasome system, mediates PD-L1 degradation and reduces its surface abundance (6–8). Targeting this degradative route thus represents a promising strategy to overcome PD-L1-mediated immune suppression in TNBC. Notably, the PI3K-Akt pathway, which is frequently hyperactivated in TNBC and contributes to tumor progression and immune evasion, serves as a critical upstream regulator linking PD-L1 expression and autophagy. Activated PI3K-Akt signaling not only upregulates PD-L1 transcription through downstream effectors including nuclear factor-κB and hypoxia-inducible factor-1α, but also participates in PD-L1 post-translational regulation (9, 10). As a canonical negative regulator of autophagy, the PI3K-Akt pathway suppresses autophagic flux and lysosomal degradation (11). However, whether and how the PI3K-Akt pathway modulates autophagy-lysosome-dependent PD-L1 degradation in TNBC remains largely unknown.

Berberine (BBR), a natural isoquinoline alkaloid primarily extracted from traditional medicinal herbs including Coptis chinensis and Phellodendron amurense, has been widely utilized in traditional medicine for centuries to treat inflammation, infection, and metabolic disorders (12, 13). Modern pharmacological studies have further validated BBR’s favorable safety profile and multi-target therapeutic properties, with prominent anti-inflammatory, anti-oxidative, and broad-spectrum anti-tumor activities (14, 15). Distinct from conventional chemotherapeutic agents, BBR exhibits selective tumor-suppressive effects with minimal cytotoxicity toward normal human cells, rendering it an ideal candidate for adjuvant tumor immunotherapy (16). Emerging evidence has demonstrated that BBR can modulate PD-L1 expression and trigger anti-tumor immune responses in multiple cancer types (17, 18). Nevertheless, the specific mechanism by which BBR regulates PD-L1 post-translational degradation, as well as its regulatory effect on immunogenic cell death (ICD) in TNBC, remains uncharacterized. ICD is a unique immunostimulatory cell death modality that reverses tumor immunosuppression by releasing damage-associated molecular patterns (DAMPs) and exposing surface “eat-me” signals, thereby awakening systemic anti-tumor immunity (19).

Based on the above research background, we hypothesized that BBR exerts anti-tumor immunomodulatory effects in TNBC by inhibiting the PI3K-Akt pathway, subsequently activating autophagy-lysosomal cascade to promote PD-L1 protein degradation, while simultaneously inducing ICD to enhance tumor immunogenicity. In the present study, we systematically demonstrate that BBR downregulates PD-L1 in a time- and dose-dependent manner and blunts interferon-γ-induced PD-L1 upregulation in TNBC cells. Mechanistically, BBR suppresses PI3K-Akt signaling to restore autophagic flux, thereby facilitating lysosome-dependent PD-L1 degradation. Moreover, BBR triggers robust ICD responses, characterized by increased calreticulin exposure and elevated HMGB1 and HSP70 release. Consistent with *in vitro* findings, *in vivo* experiments using immunocompetent 4T1 tumor-bearing mice verify that BBR inhibits TNBC growth, enhances CD8^+^ cytotoxic T cell infiltration and activation, and reduces immunosuppressive Treg accumulation, ultimately remodeling the immunosuppressive TME.

Collectively, our findings reveal that BBR triggers PD-L1 degradation through the PI3K-Akt–autophagy-lysosome axis and enhances ICD in TNBC, thereby boosting anti-tumor immunity. This study provides a mechanistic rationale for developing BBR as an adjuvant agent to improve immunotherapy outcomes in TNBC.

## 2 Materials and methods

### 2.1 Cell culture

Human TNBC cell lines (MDA-MB-231, BT-549), mouse TNBC 4T1 cells, and normal mammary epithelial MCF-10A cells were obtained from Procell Life Science & Technology. All human cell lines were authenticated by STR profiling, and 4T1 cells were verified by morphology and function. MDA-MB-231 cells were cultured in DMEM supplemented with 10% FBS and 1% penicillin-streptomycin. BT-549 cells were maintained in RPMI-1640 containing 10% FBS, 10 μg/mL insulin, and 1% penicillin-streptomycin. 4T1 cells were cultured in RPMI-1640 with 10% FBS and 1% penicillin-streptomycin, and MCF-10A cells were cultured in MCF-10A Cell Complete Medium (CM-0525). All cells were cultured at 37 °C with 5% CO_2_. All cell lines were tested negative for mycoplasma contamination by Hoechst 33258 staining and used within a low passage range.

### 2.2 Reagents and antibodies

Berberine (BBR, purity ≥ 99%, CAS No.: 2086-83-1) was dissolved in dimethyl sulfoxide (DMSO) to prepare a 100 mmol/L stock solution and stored at −20 °C in small aliquots to avoid repeated freeze-thaw cycles. The working concentration of 10 μmol/L BBR was obtained by diluting the stock solution 10,000-fold, resulting in a final DMSO concentration of 0.01% (v/v), which is well below the commonly accepted safe threshold for *in vitro* cell culture applications (20, 21). A vehicle control containing 0.01% DMSO was included in all experiments. The concentrations of other reagents (740 Y-P, LY294002, bafilomycin A1, MG132, chloroquine, 3-methyladenine, CHX, rapamycin) were determined by CCK-8 assays to ensure they reached the recommended minimal effective dose without compromising cell viability (**Supplementary Figure 1**). Key antibodies and reagents are listed in **Supplementary Table 1**.

### 2.3 Cell viability assay

Cell viability was measured using CCK-8 assay. Cells in logarithmic growth phase were harvested, and single-cell suspensions were prepared. Cells were seeded in 96-well plates at a density of 5 × 10^3^ cells per well in 100 μL of culture medium and allowed to adhere overnight. The following day, cells were treated with gradient concentrations of BBR for 12, 24, or 48 h. After incubation with CCK-8 solution for 2 h, absorbance at 450 nm was detected using a microplate reader. Relative viability was normalized to the vehicle control group. All experiments were performed in triplicate. The relative cell viability (%) for each BBR-treated well was then calculated as:


$$\text{Cell viability}\left(\%\right)=\frac{{\text{Absorbance}}_{\left(\text{treated, blank-subtracted}\right)}}{{\text{Absorbance}}_{\left(\text{control, blank-subtracted}\right)}}\times 100\%$$

### 2.4 Total protein measurement

To exclude non-specific protein loss as a confounding factor, total protein concentration was measured using the BCA assay. MDA-MB-231 and BT-549 cells were seeded at 5 × 10^5^ cells per well in 6-well plates and treated with gradient concentrations of BBR (0-10 μmol/L) or vehicle DMSO for 24 h. After treatment, cells from each well were gently trypsinized, counted, and equal numbers of viable cells were collected. Cells were lysed in RIPA buffer, and protein concentrations were determined using the BCA Protein Assay Kit according to the manufacturer’s protocol.

### 2.5 ELISA assay

PD-L1 levels in cell culture supernatants were measured using a commercial ELISA kit according to the manufacturer’s instructions. For exploratory clinical analysis, serum and tissue specimens were collected under approval from the Ethics Committee of Wuming Hospital Affiliated to Guangxi Medical University (No. WM-2026(22)) and assayed in a double-blinded manner. Detailed clinical characteristics and inclusion/exclusion criteria are fully documented in **Supplementary Table 2**.

### 2.6 Western blot assay

Total protein was extracted using RIPA lysis buffer with protease and phosphatase inhibitors. Equal amounts of protein were separated by SDS-PAGE and transferred to PVDF membranes. After blocking with 5% non-fat milk, membranes were incubated with primary antibodies at 4 °C overnight, followed by HRP-conjugated secondary antibodies at room temperature. Protein bands were visualized using ECL reagent and quantified with ImageJ software. GAPDH or β-actin served as loading controls for total and cytoplasmic proteins, and Na^+^/K^+^-ATPase for membrane proteins.

### 2.7 Cellular immunofluorescence assay

Cells were seeded onto coverslips in 24-well plates at 2 × 10^5^ cells/well and cultured overnight. After treatment, cells were fixed with 4% paraformaldehyde (15 min), permeabilized with 0.5% Triton X-100 (10 min), and blocked with 5% BSA (30 min). Primary antibodies (1:200) were applied overnight at 4 °C, followed by fluorophore-conjugated secondary antibodies (1:500, 1 h, room temperature). Nuclei were stained with DAPI or Hoechst 33342. Images were captured and fluorescence intensity was quantified using ImageJ.

### 2.8 Flow cytometry

Flow cytometry was performed on a Beckman cytometer and analyzed using FlowJo v10.8.1 software. For detection of membrane PD-L1 and CD44 expression, cells (1 × 10^6^ per sample) were washed twice with PBS, resuspended in 100 μL of PBS containing 1% FBS to minimize non-specific binding, and incubated with PE-conjugated anti-PD-L1, anti-CD44, or PE-conjugated mouse IgG2b isotype control antibodies (1:100 dilution) for 30 min at 4°C in the dark. Directly PE-conjugated primary antibodies were used throughout to avoid Fc-mediated non-specific binding via secondary reagents. After staining, cells were washed twice with PBS and resuspended in 300 μL of PBS/1% FBS for immediate acquisition. The mean fluorescence intensity (MFI) of each surface marker was quantified after subtracting the corresponding isotype control background. For tumor-infiltrating lymphocyte (TIL) analysis, tumor tissues were mechanically and enzymatically digested into single-cell suspensions, and cells were stained for viability, surface markers (CD3, CD8, CD25, CD69), and intracellular markers (Foxp3, Granzyme B). Detailed antibody information and gating strategies are provided in **Supplementary Table 3** and Supplementary Materials and Methods.

### 2.9 PD-1/PD-L1 binding assay

Cells were seeded in 24-well plates at 2 × 10^5^ cells/well and cultured overnight. After BBR treatment, cells were incubated with Alexa Fluor 488-labeled recombinant human PD-1 protein (Sino Biological, 1:100) at 37 °C for 1 h, washed, fixed with 4% paraformaldehyde (15 min), and counterstained with DAPI. Fluorescence images were captured under a fluorescence microscope (Olympus). The green fluorescence intensity, reflecting PD-L1/PD-1 binding, was quantified using ImageJ software.

### 2.10 Cycloheximide pulse-chase assay

After BBR treatment, cells were incubated with 50 μg/mL CHX and collected at 0, 1, 3, 6, 9, and 12 h. PD-L1 protein levels were detected by WB and quantified.

### 2.11 Lysosomal function assessment

Lysosomal acidification and membrane stability were evaluated using Lyso-Tracker Red and acridine orange staining respectively. Fluorescence was observed under a fluorescence microscope.

### 2.12 T cell-mediated tumor cell killing assay

Human PBMCs were isolated from fresh whole blood of healthy donors (Ethics approval No. WM-2026(22)) and activated with plate-bound anti-CD3 (1 μg/mL), soluble anti-CD28 (0.5 μg/mL), and recombinant human IL-2 (50 U/mL) for 72 h. Activated effector cells were co-cultured with BBR-pretreated TNBC cells at an effector-to-target (E:T) ratio of 5:1. Cytotoxicity was assessed by LDH release, Calcein-AM release, CCK-8, and crystal violet staining assays. Detailed experimental grouping and procedures are provided in Supplementary Materials and Methods.

### 2.13 Animal experiments

All animal procedures were approved by the Experimental Animal Welfare and Ethics Committee of Guangxi Medical University (No. 202505006). Female BALB/c nude mice (MDA-MB-231 xenografts) and BALB/c mice (4T1 syngeneic model) were housed in SPF conditions. Mice were randomly divided into control, low-dose BBR (4 mg/kg), and high-dose BBR (8 mg/kg) groups (n=6 per group). BBR or vehicle was administered daily by intraperitoneal injection. Tumor volume and body weight were measured every 2 days. Tumor volume was calculated as 1/2 × length × width^2^. Mice were euthanized at the ethical endpoint, and tumor tissues were harvested for subsequent analysis.

### 2.14 Immunohistochemistry assay

Paraffin sections were deparaffinized, blocked, and incubated with primary antibodies at 4 °C overnight, followed by secondary antibodies. Staining was visualized with DAB and counterstained with hematoxylin. Positive cells were quantified using ImageJ in a blinded manner.

### 2.15 Statistical analysis

All data are presented as mean ± standard error of the mean (SEM) from at least three independent experiments. Statistical analysis was performed using GraphPad Prism 9.5. Differences between two groups were analyzed by unpaired Student’s t-test. Multiple groups were compared using one-way ANOVA followed by Dunnett’s or Tukey’s post-hoc test. Tumor growth curves were analyzed by two-way repeated-measures ANOVA with Bonferroni correction. *P* < 0.05 was considered statistically significant.

## 3 Results

### 3.1 BBR regulates PD-L1 expression in triple-negative breast cancer cells

#### 3.1.1 BBR downregulates PD-L1 protein expression in TNBC cells in a time- and dose-dependent manner

As an exploratory pilot analysis in a small cohort (serum: n=4 vs 4; tissue: n=3 paired) using ELISA for serum samples and Western blot for tumor tissues, we observed higher PD-L1 abundance in serum and tumor tissues from breast cancer patients relative to normal controls (**Supplementary Figures 3A, B**; **Supplementary Table 2**). Of note, this preliminary trend requires further validation in larger patient cohorts due to the limited sample size and inherent tumor heterogeneity.

**Figure 1:**
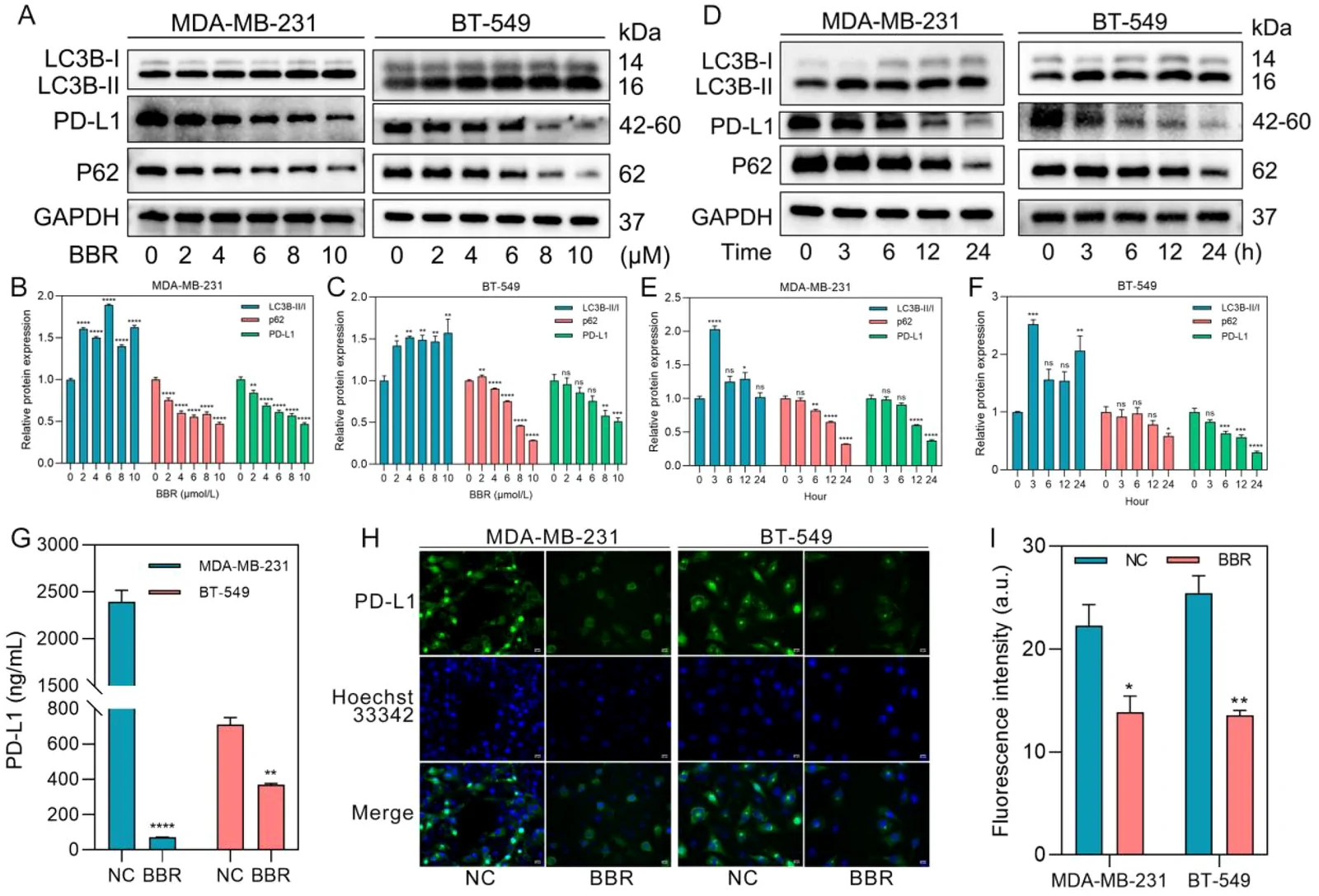
BBR downregulates PD‑L1 expression and induces autophagy in TNBC cells. **(A)** WB analysis of PD-L1, LC3B, and P62 protein levels in MDA-MB-231 and BT-549 cells treated with gradient concentrations of BBR (0, 2, 4, 6, 8, 10 μmol/L) for 24 h. **(B, C)** Quantitative quantification of relative protein expression in panel **(A)**. **(D)** WB detection of PD-L1, LC3B, and P62 expression in TNBC cells treated with 10 μmol/L BBR at indicated time points (0, 3, 6, 12, 24 h). **(E, F)** Quantitative analysis of relative protein levels in panel D. GAPDH was used as the internal loading control for **(A)** and **(D)**. **(G)** ELISA detection of soluble PD‑L1 in cell culture supernatants. **(H)** Representative immunofluorescence images of PD‑L1 (green) in control and BBR‑treated TNBC cells; nuclei were stained with Hoechst 33342 (scale bar = 20 μm). **(I)** Quantitative analysis of PD‑L1 fluorescence intensity in the corresponding groups. Data are presented as mean ± SEM. ns, not significant. *P<0.05, **P<0.01, ***P<0.001, ****P<0.0001 vs. the corresponding control group.

We next explored the regulatory effect of BBR on PD-L1 protein expression in two TNBC cell lines (MDA-MB-231 and BT-549). Western blot analysis showed that BBR reduced PD-L1 expression in a dose-dependent manner within the concentration range of 0 - 10 μmol/L, with 10 μmol/L BBR producing the maximal inhibitory effect after 24 h of treatment (**Figures 1A–C**). Similarly, BBR suppressed PD-L1 protein levels in a time-dependent fashion, with the most pronounced reduction observed at 24 h (**Figures 1D–F**). To investigate whether BBR induces PD-L1 degradation through autophagy activation, we examined key autophagy markers. BBR treatment dose-dependently increased the LC3B-II/I ratio, an indicator of autophagosome formation, in both MDA-MB-231 and BT-549 cells, while concurrently decreasing P62 levels, reflecting enhanced autophagic flux (**Figures 1A–C**). Time-course analysis confirmed these effects peaked at 24 h (**Figures 1D–F**). These results demonstrate that BBR activates autophagy in TNBC cells, supporting our hypothesis that autophagy activation contributes to PD-L1 degradation via the autophagy-lysosome pathway.

Subsequent validation experiments excluded non-specific cytotoxicity and global protein suppression as potential confounding factors. CCK-8 assays confirmed that 10 μmol/L BBR did not significantly affect the viability of TNBC cells following 24 h incubation (**Supplementary Figures 2E, F**). No apparent changes in total protein content or cell apoptosis were detected under the same treatment conditions (**Supplementary Figures 3C–G**). Furthermore, this therapeutic concentration exhibited minimal cytotoxicity toward normal MCF-10A mammary epithelial cells (Supplementary **Supplementary Figures 2D, H, L**). Collectively, these results verify that BBR specifically inhibits PD-L1 expression rather than inducing generalized cellular damage. Therefore, 10 μmol/L BBR with a 24 h treatment duration was selected for all subsequent mechanistic investigations.

#### 3.1.2 BBR reduces total expression and membrane distribution of PD-L1

Following the confirmation of BBR-mediated downregulation of total PD-L1 protein, we further investigated the effect of BBR on the subcellular distribution of PD-L1 in TNBC cells. Consistent with the immunoblotting results, ELISA quantification showed a marked reduction in soluble PD-L1 levels in the supernatant of BBR-treated TNBC cells (**Figure 1G**). Immunofluorescence staining revealed that both MDA-MB-231 and BT-549 cells exhibited strong green PD-L1 fluorescence in the control group, with signals localized at both the plasma membrane and the cytoplasm, consistent with high basal PD-L1 expression. Following BBR treatment (10 μmol/L, 24 h), PD-L1 fluorescence was markedly attenuated at both membrane and cytoplasmic compartments in both cell lines (**Figures 1H, I**). The concurrent reduction of membrane-associated and cytoplasmic PD-L1 signals indicates a global downregulation rather than subcellular redistribution.

**Figure 2:**
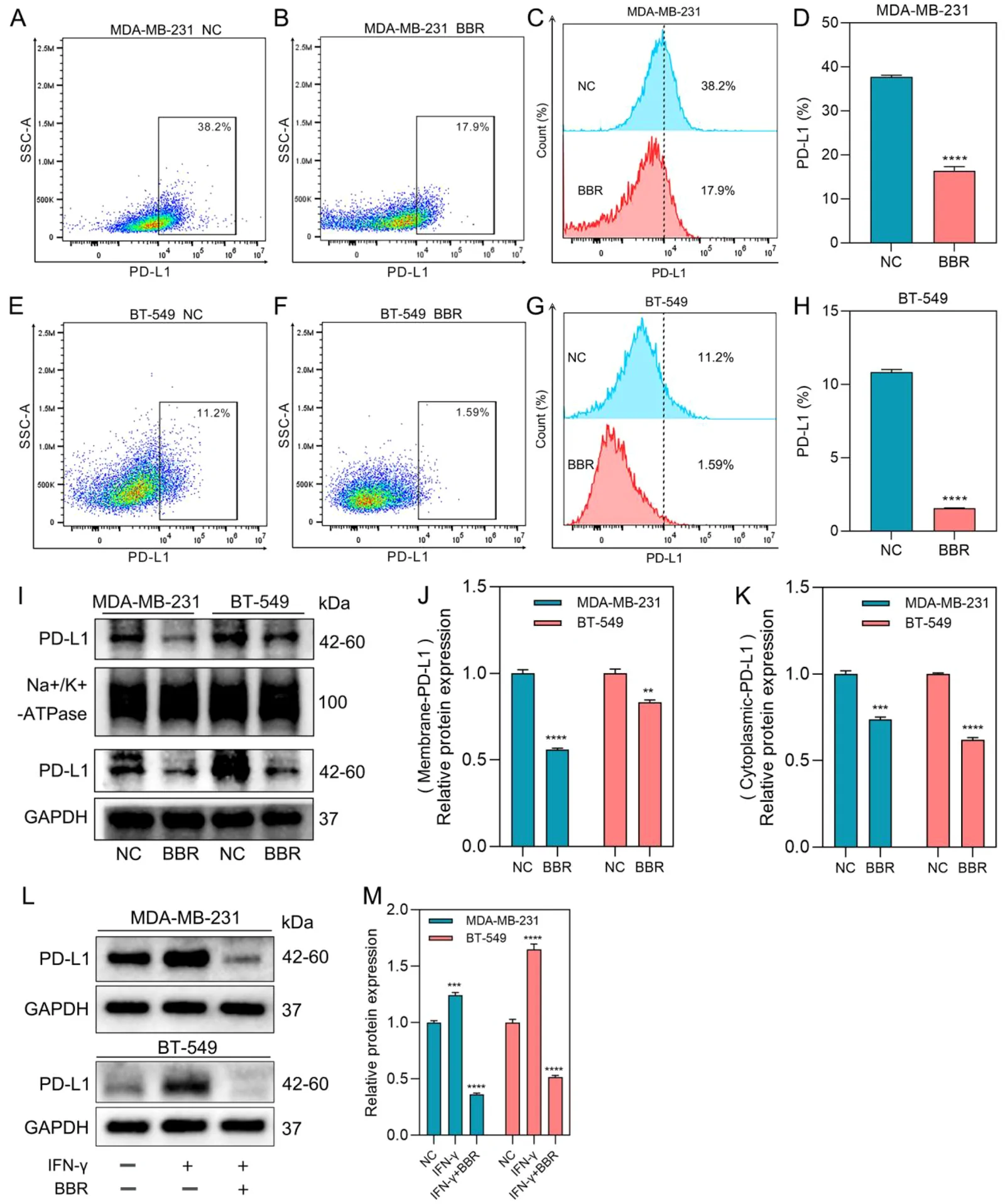
BBR decreases membrane PD-L1 abundance and blocks IFN-γ-mediated PD‑L1 upregulation in TNBC cells. **(A–H)** Flow cytometry detection of membrane PD-L1 expression in TNBC cells. Representative histograms and statistical analysis of PD‑L1-positive cell proportions with or without BBR intervention. **(I)** WB analysis of PD-L1 expression in membrane and cytoplasmic protein fractions. Na^+^/K^+^-ATPase and GAPDH were used as internal controls for membrane and cytoplasmic proteins, respectively. **(J, K)** Quantitative analysis of relative PD‑L1 levels in membrane and cytoplasmic fractions corresponding to panel **(I)**. **(L, M)** WB analysis of PD-L1 expression in TNBC cells stimulated with 5 ng/mL IFN‑γ alone or following BBR pretreatment; GAPDH was used as the internal loading control. Data are presented as mean ± SEM. ns, not significant. *P<0.05, **P<0.01, ***P<0.001, ****P<0.0001 vs. the corresponding control group.

Cell membrane-localized PD-L1 is a critical regulator of tumor immune escape by binding to T-cell surface PD-1. Flow cytometry analysis revealed a significant reduction in surface PD-L1 fluorescence intensity in BBR-treated MDA-MB-231 (**Figures 2A–D**) and BT-549 cells, indicating that BBR diminishes membrane PD-L1 distribution (**Figures 2E–H**). Cell fractionation and Western blot assays further demonstrated that BBR decreased PD-L1 protein levels in both cytoplasmic and membrane fractions (**Figures 2I–K**).

The expression of the membrane-specific marker Na^+^/K^+^-ATPase remained unchanged after BBR treatment, excluding non-specific damage to cell membrane structure. Additionally, no significant difference in the surface biomarker CD44 was observed between the control and BBR groups (**Supplementary Figures 3H–K**). Collectively, these findings indicate that BBR specifically suppresses PD-L1 expression and membrane localization, rather than inducing a global downregulation of membrane proteins in TNBC cells.

#### 3.1.3 BBR suppresses IFN-γ-mediated PD-L1 upregulation

Interferon-γ (IFN-γ) is a pivotal inflammatory cytokine abundant in the tumor microenvironment, which potently induces PD-L1 expression and facilitates tumor immune escape. We further investigated whether BBR could counteract IFN-γ-triggered PD-L1 upregulation in TNBC cells. TNBC cells were stimulated with 5 ng/mL IFN-γ for 24 h to mimic the inflammatory tumor microenvironment. Western blot results confirmed that IFN-γ stimulation markedly elevated endogenous PD-L1 protein levels. Notably, 2 h of BBR pretreatment efficiently reversed IFN-γ-induced PD-L1 accumulation in TNBC cells (**Figures 2L, M**). These data demonstrate that BBR is capable of suppressing PD-L1 overexpression even under inflammatory conditions, suggesting its potential to remodel the immunosuppressive tumor microenvironment.

### 3.2 Anti-tumor immune regulatory effects of BBR

#### 3.2.1 BBR restricts tumor immune escape and enhances immune cell cytotoxicity

To establish a stable and efficient immune co-culture system, we first optimized the effector-to-target (E:T) cell ratio for subsequent functional assays. CCK-8 results showed that the cytotoxic effect of peripheral blood mononuclear cells (PBMCs) on TNBC cells increased in an E:T ratio-dependent manner, with significant tumor cell killing observed at ratios of 5:1, 10:1, and 15:1 (**Supplementary Figures 3L, M**). Considering experimental stability and operability, the E:T ratio of 5:1 was selected for all subsequent co-culture experiments, consistent with previously reported studies (22–24).

We next evaluated the biosafety of the experimental BBR concentration on PBMCs. CCK-8 detection demonstrated that BBR treatment at the working concentration exerted no obvious cytotoxicity to PBMCs. After 24 h of incubation with 10 μmol/L BBR, PBMC viability remained above 80% with no significant reduction relative to the control group (**Supplementary Figures 3N, O**), verifying the safety of this concentration for immune cell functional analysis.

We further assessed whether BBR could restore immune-mediated tumor killing under IFN-γ-induced immunosuppressive conditions. Both CCK-8 quantitative analysis and crystal violet staining confirmed that BBR pretreatment significantly enhanced PBMC-dependent cytotoxicity against TNBC cells (**Supplementary Figures 2A–C**). The combined BBR and PBMC treatment group exhibited markedly reduced tumor cell viability, indicating that BBR effectively alleviates TNBC immune escape and potentiates anti-tumor immune responses.

#### 3.2.2 BBR blocks PD-1/PD-L1 interaction and strengthens T cell anti-tumor cytotoxicity

**Figure 3:**
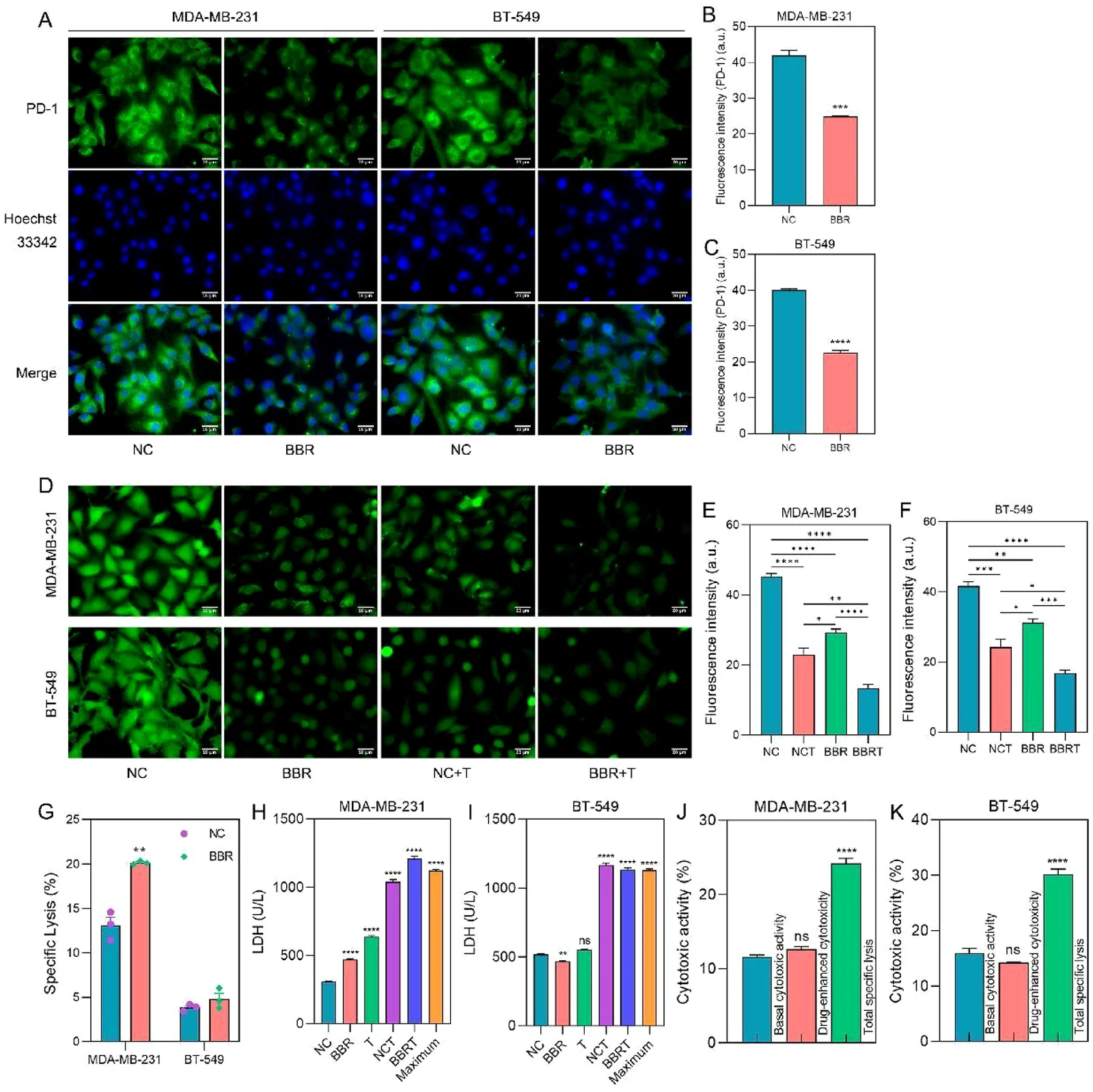
BBR suppresses PD‑L1/PD-1 interaction and potentiates T cell‑driven cytotoxicity toward TNBC cells. **(A)** Representative immunofluorescence images of Alexa Fluor 488-conjugated PD-1 binding to control and BBR‑treated TNBC cells. Nuclei were counterstained with Hoechst 33342; scale bar = 20 μm. **(B, C)** Quantitative analysis of PD0-1 fluorescence intensity in MDA-MB-231 and BT-549 cells, reflecting the relative PD-L1/PD-1 binding capacity. **(D)** Representative Calcein-AM staining of viable TNBC cells following co-culture with activated T cells under different treatment conditions; scale bar = 20 μm. **(E, F)** Quantitative analysis of Calcein-AM fluorescence intensity in the two cell lines. **(G)** Calculated specific lysis rate of TNBC cells based on Calcein-AM release after T cell‑mediated cytotoxicity. **(H, I)** LDH release assay detecting T cell‑mediated cytotoxicity against TNBC cells under designated experimental groups, including blank control, single treatment, and maximum lysis induced by Triton X-100. **(J, K)** Quantitative analysis of T cell‑mediated cytotoxicity against MDA-MB-231 and BT-549 cells, including basal T cell killing activity, BBR‑enhanced killing activity, and maximum release activity (calculated as: (experimental release – background release) / (maximum release – background release) × 100%) (n = 3 independent experiments for each assay). Data are presented as mean ± SEM. ns, not significant. *P < 0.05, **P < 0.01, ***P < 0.001, ****P < 0.0001 vs. the corresponding control group.

Given the inhibitory effect of BBR on membrane PD-L1 expression, we further explored whether BBR interferes with the PD-1/PD-L1 immune checkpoint interaction. An immunofluorescence binding assay using Alexa Fluor 488-conjugated PD-1 recombinant protein was performed. Quantitative fluorescence analysis revealed a significant reduction in PD-1 binding capacity on the surface of BBR-treated TNBC cells (**Figures 3A–C**), suggesting that BBR efficiently disrupts PD-1/PD-L1 ligation.

Calcein-AM staining was subsequently applied to evaluate T cell-mediated tumor cytotoxicity. After co-culture with activated T cells, TNBC cells in the BBR group displayed a decreased number of viable cells and an increased tumor lysis rate (**Figures 3D–G**). An LDH release assay was further employed to validate tumor cell injury. The elevated LDH release in the BBR treatment group confirmed enhanced T cell-mediated cytotoxicity. Collectively, these results demonstrate that BBR blocks PD-1/PD-L1 binding and thereby reinforces T cell-dependent anti-tumor immune killing (**Figures 3H–K**).

#### 3.2.3 *In vivo* efficacy and immune microenvironment modulation of BBR

##### 3.2.3.1 Inhibitory effect and biosafety of BBR on TNBC xenograft growth

**Figure 4:**
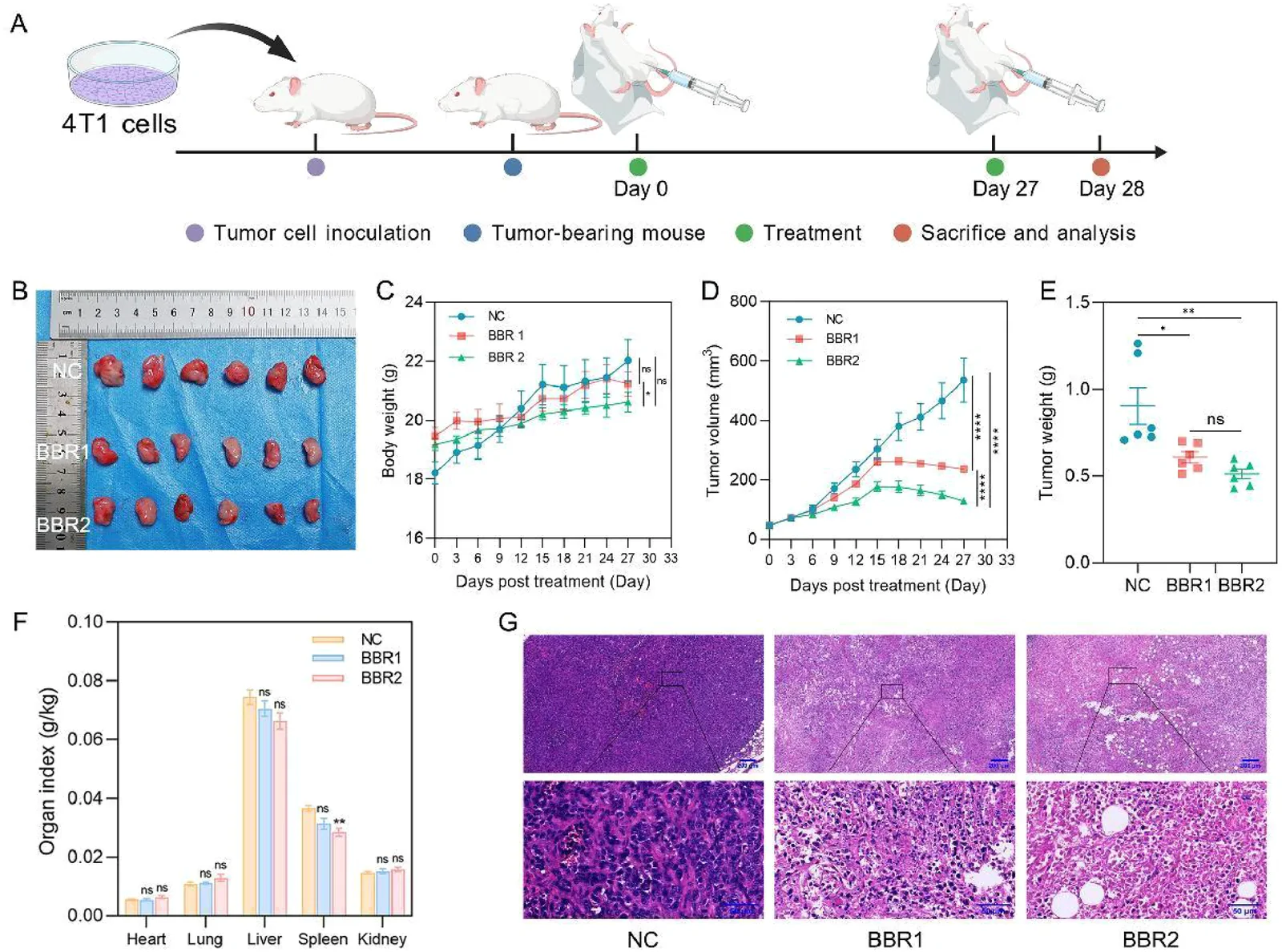
BBR suppresses tumor growth with a favorable in vivo safety profile in immunocompetent 4T1 tumor‑bearing mice. **(A)** Schematic overview of the 4T1 orthotopic mouse model and experimental workflow. **(B)** Representative macroscopic images of 4T1 tumors from control and BBR‑treated mice (NC: control, BBR1: 4 mg/kg BBR, BBR2: 8 mg/kg BBR). **(C)** Body weight dynamics of mice in each group during treatment. **(D)** Tumor growth curves recorded throughout the experimental period. **(E)** Final tumor weight of mice in different treatment groups. **(F)** Organ indices of major visceral organs, including the heart, lung, liver, spleen and kidney. **(G)** H&E staining of 4T1 tumor tissues at low and high magnification (scale bars = 200 μm and 50 μm); necrotic regions are marked (top panels: low magnification; bottom panels: high magnification) (n = 6 mice per group). Data are presented as mean ± SEM. ns, not significant. *P < 0.05, **P < 0.01, ***P < 0.001, ****P < 0.0001 vs. the corresponding control group.

We further established *in vivo* xenograft models to evaluate the anti-tumor efficacy and systemic biosafety of BBR. No obvious tumor suppressive effect was observed in nude mouse xenografts (**Supplementary Figures 4D–G**). In contrast, in immunocompetent BALB/c mice bearing 4T1 orthotopic tumors, continuous 28-day BBR treatment significantly inhibited tumor growth. Both tumor volume and final tumor weight were reduced in mice treated with 4 mg/kg and 8 mg/kg BBR, exhibiting a clear dose-dependent anti-tumor effect (**Figures 4A–E**).

No abnormal physiological behaviors or body weight loss were observed in any experimental group. Additionally, no significant differences in major visceral organ indices were detected among groups. Histopathological examination revealed no obvious pathological damage to vital organs, confirming the low systemic toxicity of BBR *in vivo* (**Figure 4H**; **Supplementary Figure 4H**). Moreover, HE staining of tumor tissues showed enlarged necrotic areas in BBR-treated mice, further validating the inhibitory effect of BBR on TNBC tumor progression (**Figure 4G**).

##### 3.2.3.2 Remodeling of tumor immune microenvironment induced by BBR

Immunohistochemistry and Western blot assays were performed to assess the *in vivo* effects of BBR on tumor proliferation, autophagy, immune checkpoint expression, and immunogenic cell death (ICD). Flow cytometry was further used to quantify the infiltration and activation status of tumor-infiltrating lymphocytes (TILs).

**Figure 5:**
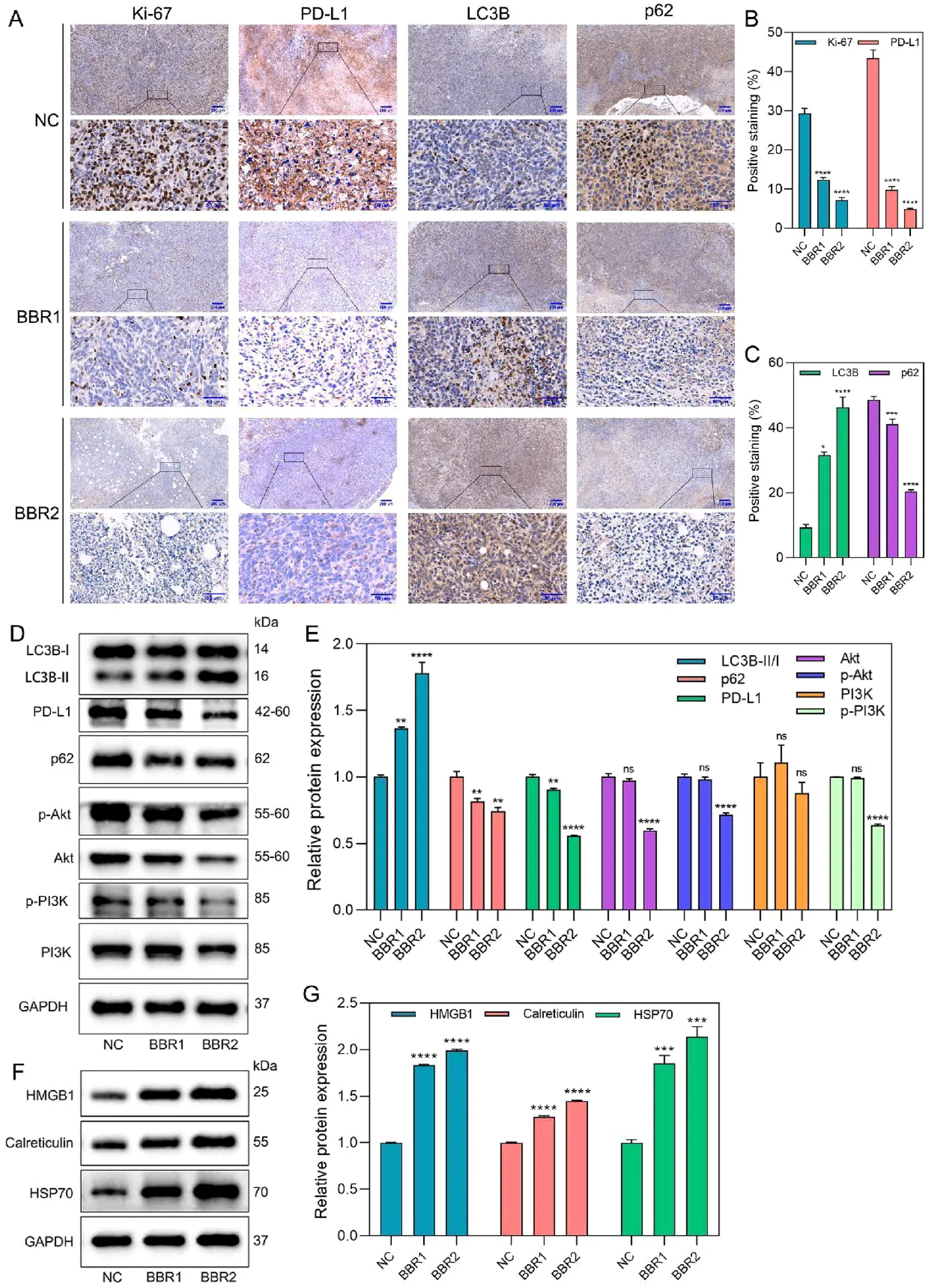
Berberine modulates relevant molecular profiles to suppress 4T1 orthotopic tumor growth. **(A)** Immunohistochemical staining of Ki-67, LC3B, P62 and PD-L1 in tumor tissues. Images were captured at low (scale bar = 200 μm) and high magnification (scale bar = 50 μm). **(B, C)** Quantitative analysis of positive staining ratios of indicated biomarkers. **(D)** WB analysis of PI3K-Akt signaling, autophagy‑related proteins and PD-L1 in tumor tissues. **(E)** Quantitative analysis of protein expression in panel **(D)**. **(F)** WB analysis of ICD‑associated proteins including HMGB1, calreticulin and HSP70. **(G)** Relative quantification of protein levels shown in panel **(F)**. Data are presented as mean ± SEM. ns, not significant. *P < 0.05, **P < 0.01, ***P < 0.001, ****P < 0.0001 vs. the corresponding control group.

Immunohistochemical analysis of tumor tissues demonstrated that BBR treatment decreased the positive rates of PD-L1 and Ki-67, increased LC3B expression, and reduced P62 levels. These results indicated that BBR suppresses tumor cell proliferation and PD-L1 expression while triggering autophagy activation *in vivo* (**Figures 5A–C**). Consistent with the histological results, Western blot analysis showed that BBR inhibited the phosphorylation of PI3K and Akt, downregulated PD-L1 and p62, and upregulated LC3B-II protein levels. These findings confirmed that BBR modulates PD-L1 expression by suppressing the PI3K-Akt signaling pathway and activating autophagy (**Figures 5D, E**). Furthermore, the protein levels of the ICD markers HMGB1, calreticulin, and HSP70 were markedly elevated in BBR-treated tumor tissues (**Figures 5F, G**).

**Figure 6:**
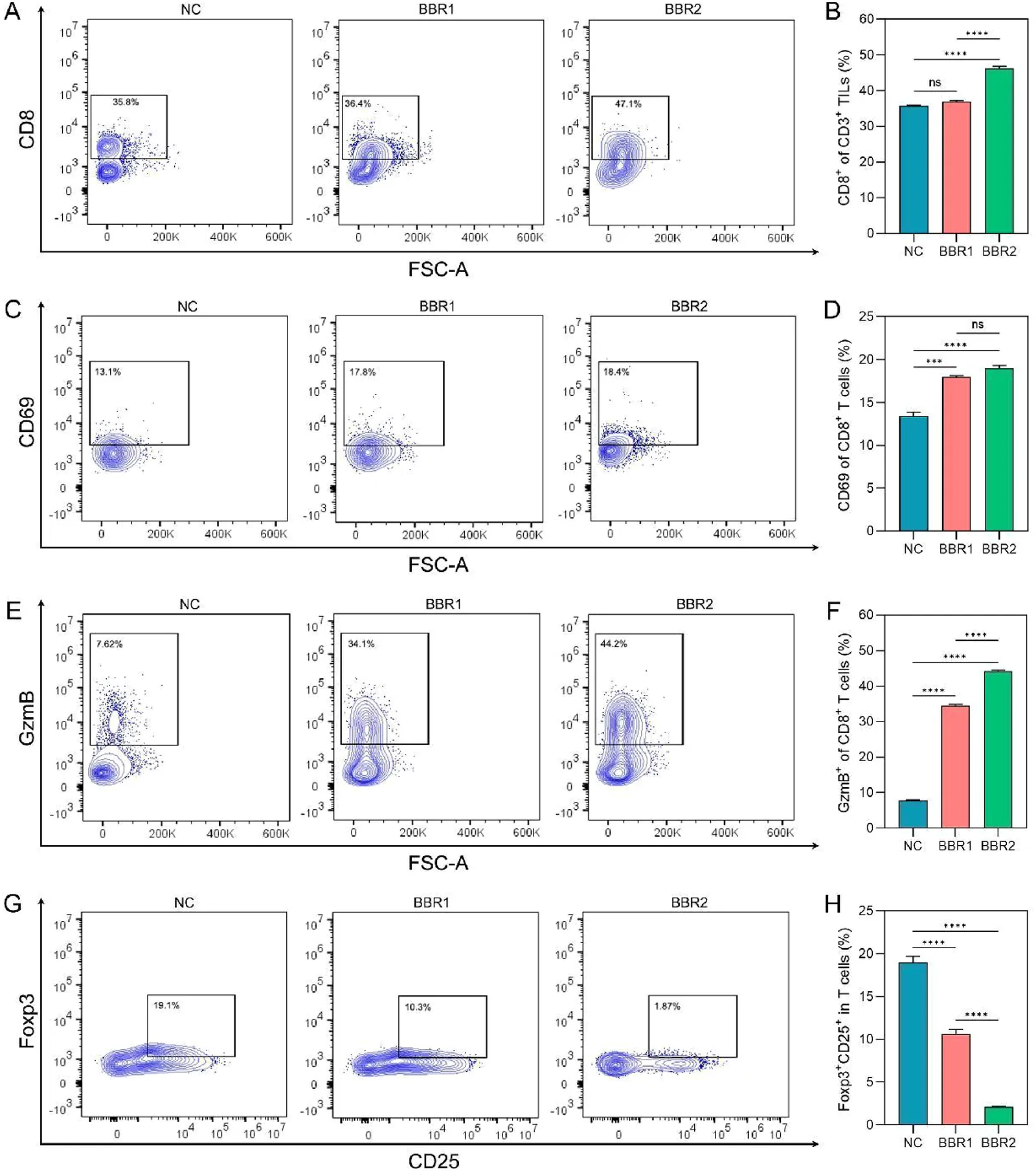
BBR reprograms the intratumoral T cell landscape in the 4T1 orthotopic tumor model. **(A, B)** Representative flow cytometry plots and quantitative analysis of intratumoral CD8^+^ T cell proportions among different groups. **(C, D)** Representative flow cytometry plots and quantitative analysis of activated CD69⁺CD8^+^ T cells in tumor tissues. **(E, F)** Representative flow cytometry plots and quantitative analysis of granzyme B^+^ cytotoxic CD8^+^ T cells in tumor tissues. **(G, H)** Representative flow cytometry plots and quantitative analysis of immunosuppressive Foxp3^+^CD25^+^ regulatory T cells within tumor tissues. Data are presented as mean ± SEM. ns, not significant. *P < 0.05, **P < 0.01, ***P < 0.001, ****P < 0.0001 vs. the corresponding control group.

Flow cytometry analysis of TILs revealed that BBR treatment increased the proportions of activated CD8^+^ T cells and granzyme B-expressing cytotoxic CD8^+^ T cells. Meanwhile, the abundance of immunosuppressive Foxp3^+^CD25^+^ regulatory T cells (Tregs) was significantly decreased (**Figures 6A–H**). Collectively, these results demonstrate that BBR remodels the tumor immune microenvironment, relieves local tumor immunosuppression, and potentiates anti-tumor immune responses.

### 3.3 Molecular mechanism underlying BBR-mediated PD-L1 regulation

#### 3.3.1 PD-L1 expression is modulated at post-translational level

**Figure 7:**
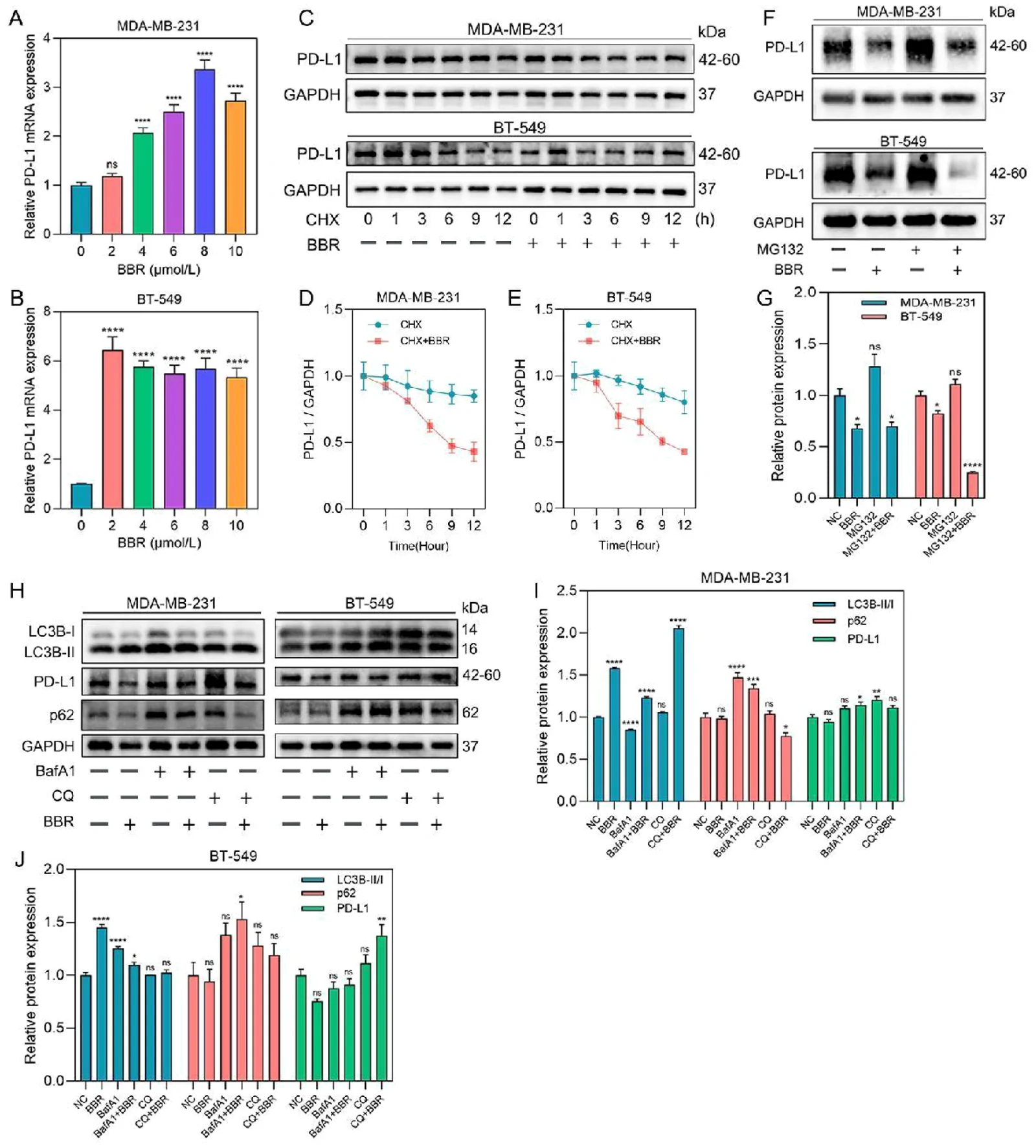
Berberine reduces PD-L1 through transcriptional inhibition and autophagy‑lysosomal degradation in TNBC cells. **(A, B)** qRT‑PCR analysis of relative PD-L1 mRNA expression in two TNBC cell lines treated with increasing concentrations of BBR for 24 h. **(C)** WB analysis of PD-L1 protein stability via CHX pulse‑chase assay in cells treated with CHX alone or combined with BBR. **(D, E)** Quantitative analysis of relative PD-L1 protein levels in two TNBC cell lines, reflecting accelerated degradation upon BBR intervention. **(F)** WB analysis of PD‑L1 expression in cells treated with BBR alone or combined with proteasome inhibitor MG132. **(G)** Quantitative analysis of PD‑L1 levels corresponding to panel **(F)**. **(H)** WB analysis of PD-L1, LC3B and P62 in cells treated with BBR together with lysosomal inhibitors BafA1 or CQ. GAPDH was used as the internal loading control. **(I, J)** Quantitative analysis of protein expression shown in panel **(H)**. Data are presented as mean ± SEM. ns, not significant. *P < 0.05, **P < 0.01, ***P < 0.001, ****P < 0.0001 vs. the corresponding control group.

To clarify the regulatory mechanism by which BBR modulates PD-L1 expression, we examined PD-L1 transcription levels and protein stability in TNBC cells. qRT-PCR analysis showed that BBR treatment slightly upregulated PD-L1 mRNA expression, which contradicted the decreased PD-L1 protein abundance (**Figures 7A,B**). This discrepancy indicated that BBR does not suppress PD-L1 transcription. CHX pulse-chase assays further demonstrated that BBR significantly shortened the half-life of PD-L1 protein, confirming that BBR facilitates PD-L1 degradation predominantly at the post-translational level (**Figures 7C–E**).

#### 3.3.2 The autophagy-lysosomal pathway mediates BBR-induced PD-L1 degradation

Pharmacological inhibition assays were performed to identify the primary pathway responsible for BBR-triggered PD-L1 degradation. Blockade of the proteasome pathway with MG132 failed to restore PD-L1 protein levels in BBR-treated TNBC cells, excluding the proteasome system as the major mechanism underlying BBR-mediated PD-L1 downregulation (**Figures 7F, G**). In contrast, inhibition of lysosomal function using BafA1 and CQ efficiently rescued PD-L1 expression, verifying that intact lysosomal activity is essential for BBR-induced PD-L1 degradation (**Figures 7H–J**).

Immunofluorescence co-localization analysis further validated the tight association between autophagic activation and PD-L1 degradation. To functionally confirm whether BBR eliminates PD-L1 through the intact autophagy-lysosomal cascade, we performed rescue experiments with two autophagy inhibitors: 3-MA to block autophagy initiation and BafA1 to inhibit late lysosomal degradation. BBR treatment reduced intracellular PD-L1 abundance while enhancing autophagic activity, whereas pharmacological blockade of autophagy at either early or late stages abolished BBR-mediated PD-L1 clearance. Detailed imaging evidence supporting BBR-induced activation of the autophagy-lysosomal cascade is provided in **Supplementary Figure 5**. Taken together, these results demonstrate that BBR promotes PD-L1 degradation via activating the complete autophagy-lysosomal signaling pathway.

#### 3.3.3 BBR maintains normal lysosomal function during PD-L1 degradation

**Figure 8:**
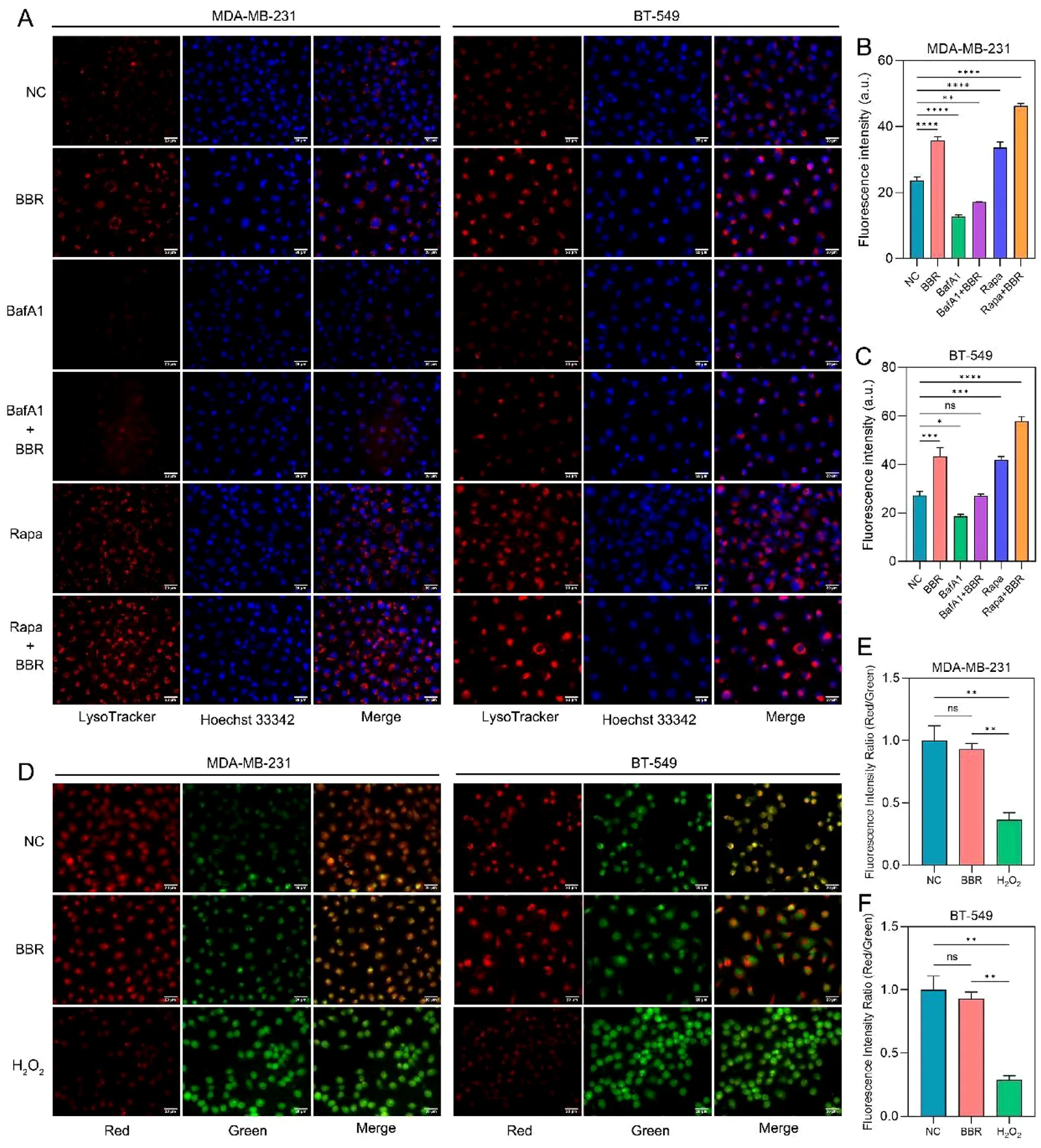
BBR enhances lysosomal accumulation while preserving lysosomal membrane integrity in TNBC cells. **(A)** LysoTracker Red staining visualizes functional acidic lysosomes in TNBC cells. Nuclei were counterstained with Hoechst 33342; scale bar = 20 μm. **(B, C)** Quantitative analysis of LysoTracker Red fluorescence intensity reflects enhanced lysosomal accumulation after BBR treatment. **(D)** Acridine orange staining assesses lysosomal membrane integrity. Red signal represents intact lysosomes, and green signal indicates lysosomal membrane damage; scale bar = 20 μm. **(E, F)** Quantitative analysis of red‑to‑green fluorescence ratio. BBR did not compromise lysosomal membrane integrity, whereas H_2_O_2_ significantly impaired membrane integrity. Data are presented as mean ± SEM. ns, not significant. *P < 0.05, **P < 0.01, ***P < 0.001, ****P < 0.0001 vs. the corresponding control group.

To exclude non-specific protein degradation caused by lysosomal damage, we further evaluated the effects of BBR on lysosomal biogenesis, activity, and membrane integrity in TNBC cells. LysoTracker Red staining revealed intensified lysosomal fluorescence signals in BBR-treated cells, indicating that BBR promotes lysosomal biogenesis and enhances lysosomal activity. Pharmacological intervention experiments showed that BafA1 pretreatment attenuated BBR-induced lysosomal activation, whereas rapamycin exerted a synergistic stimulatory effect (**Figures 8A–C**).

Acridine orange staining was performed to assess lysosomal membrane stability. The red/green fluorescence ratio showed no significant difference between the control and BBR groups, suggesting that BBR does not compromise lysosomal membrane integrity. In contrast, H_2_O_2_ treatment remarkably disrupted lysosomal structure and reduced the fluorescence ratio as a positive control. Collectively, these results demonstrate that BBR-mediated PD-L1 degradation relies on physiologically functional lysosomes rather than non-specific lysosomal damage (**Figures 8D–F**).

#### 3.3.4 BBR upregulates immunogenic cell death related biomarkers

**Figure 9:**
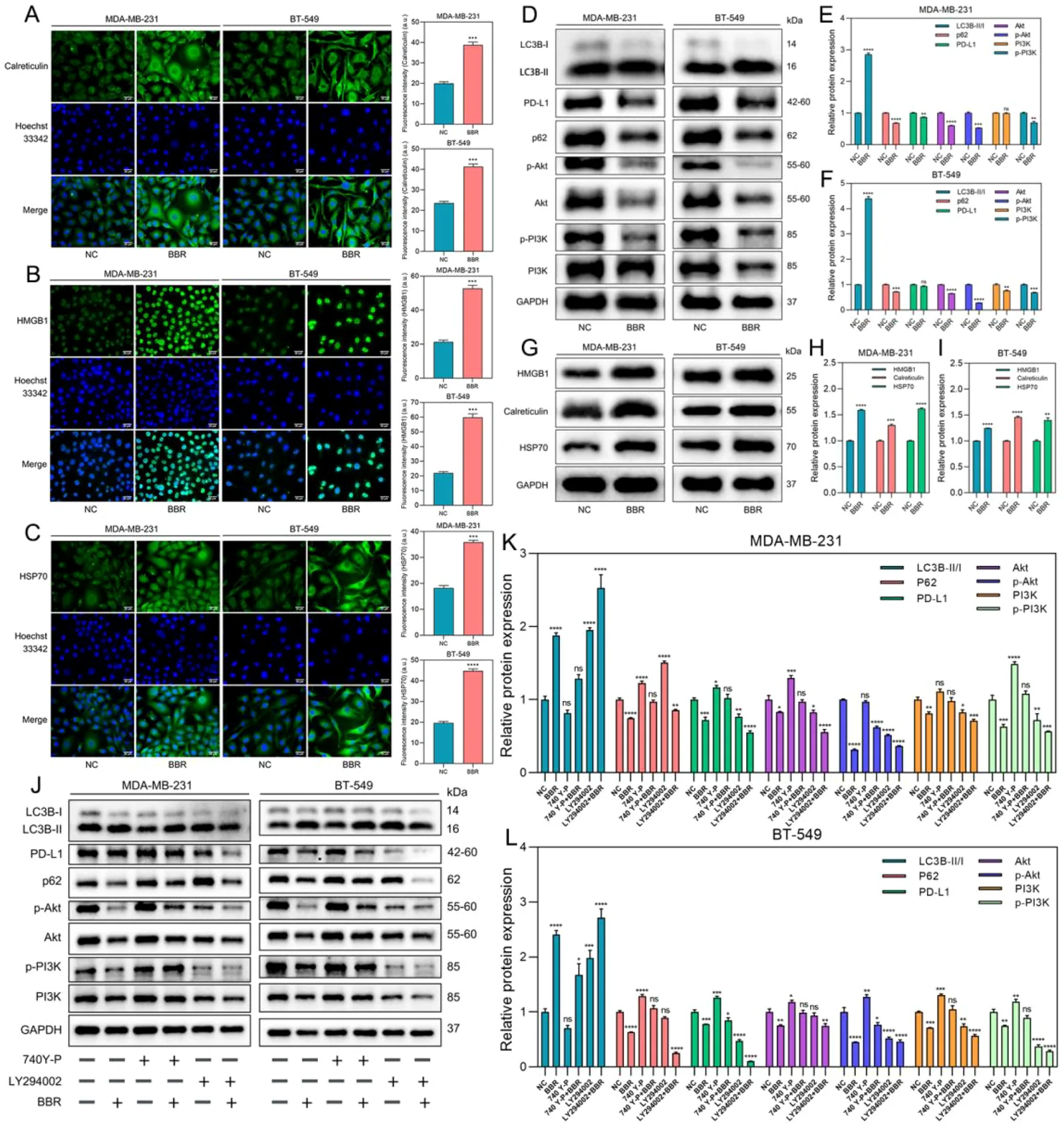
BBR elevates ICD biomarker levels and downregulates PD-L1 through PI3K-Akt pathway inhibition in TNBC cells. **(A–C)** Immunofluorescence detection of CRT, HMGB1 and HSP70 in control and BBR‑treated cells. All target proteins were labeled green, and nuclei were counterstained with Hoechst 33342 (scale bar = 20 μm). Quantitative results show enhanced expression of these ICD markers upon BBR exposure. **(D)** WB analysis of PI3K‑Akt signaling molecules, autophagy‑related proteins and PD‑L1 in cells with or without BBR treatment. GAPDH was used as the internal loading control. **(E, F)** Quantitative analysis of protein expression derived from panel **(D)**. **(G)** WB analysis of CRT, HMGB1 and HSP70 protein abundance in different groups. **(H, I)** Quantitative analysis of ICD‑related protein levels corresponding to panel **(G)**. **(J)** WB analysis of PI3K‑Akt cascade, autophagy markers and PD-L1 under single or combined treatments with BBR, the PI3K activator 740Y‑P and the inhibitor LY294002. **(K, L)** Quantitative analysis of protein expression levels in panel **(J)**. Data are presented as mean ± SEM. ns, not significant. *P < 0.05, **P < 0.01, ***P < 0.001, ****P < 0.0001 vs. the corresponding control group.

To determine whether BBR modulates tumor immunogenicity, we detected the expression of canonical immunogenic cell death (ICD) markers. Immunofluorescence staining showed elevated fluorescence intensity of CRT, HMGB1, and HSP70 following BBR treatment. Western blot quantification further confirmed the upregulated protein levels of these three ICD markers in BBR-exposed TNBC cells (**Figures 9A–C**). These data indicate that BBR effectively triggers ICD responses in TNBC cells.

#### 3.3.5 PI3K-Akt signaling governs autophagy-lysosomal PD-L1 degradation

Rescue experiments were performed to elucidate the upstream signaling mechanism by which BBR regulates autophagy-dependent PD-L1 degradation. Western blot results showed that BBR decreased the total protein and phosphorylation levels of PI3K and Akt, indicating effective suppression of PI3K-Akt signaling. In parallel, BBR treatment increased LC3B-II expression and decreased P62 levels, consistent with autophagy activation. Treatment with the PI3K activator 740 Y-P successfully reversed BBR-induced pathway inhibition, abrogated autophagy activation as evidenced by decreased LC3B-II and increased P62, and restored PD-L1 protein expression. Conversely, the PI3K inhibitor LY294002 recapitulated the inhibitory effects of BBR on PI3K-Akt signaling and PD-L1 accumulation, while also increasing LC3B-II and decreasing P62, mirroring the autophagy-activating effect of BBR. Combined treatment with BBR and LY294002 produced no additional synergistic effects (**Figures 9D–L**). In conclusion, BBR suppresses PI3K-Akt signaling, thereby triggering the autophagy-lysosomal cascade and ultimately promoting PD-L1 autophagic degradation in TNBC cells.

## 4 Discussion

**Figure 10:**
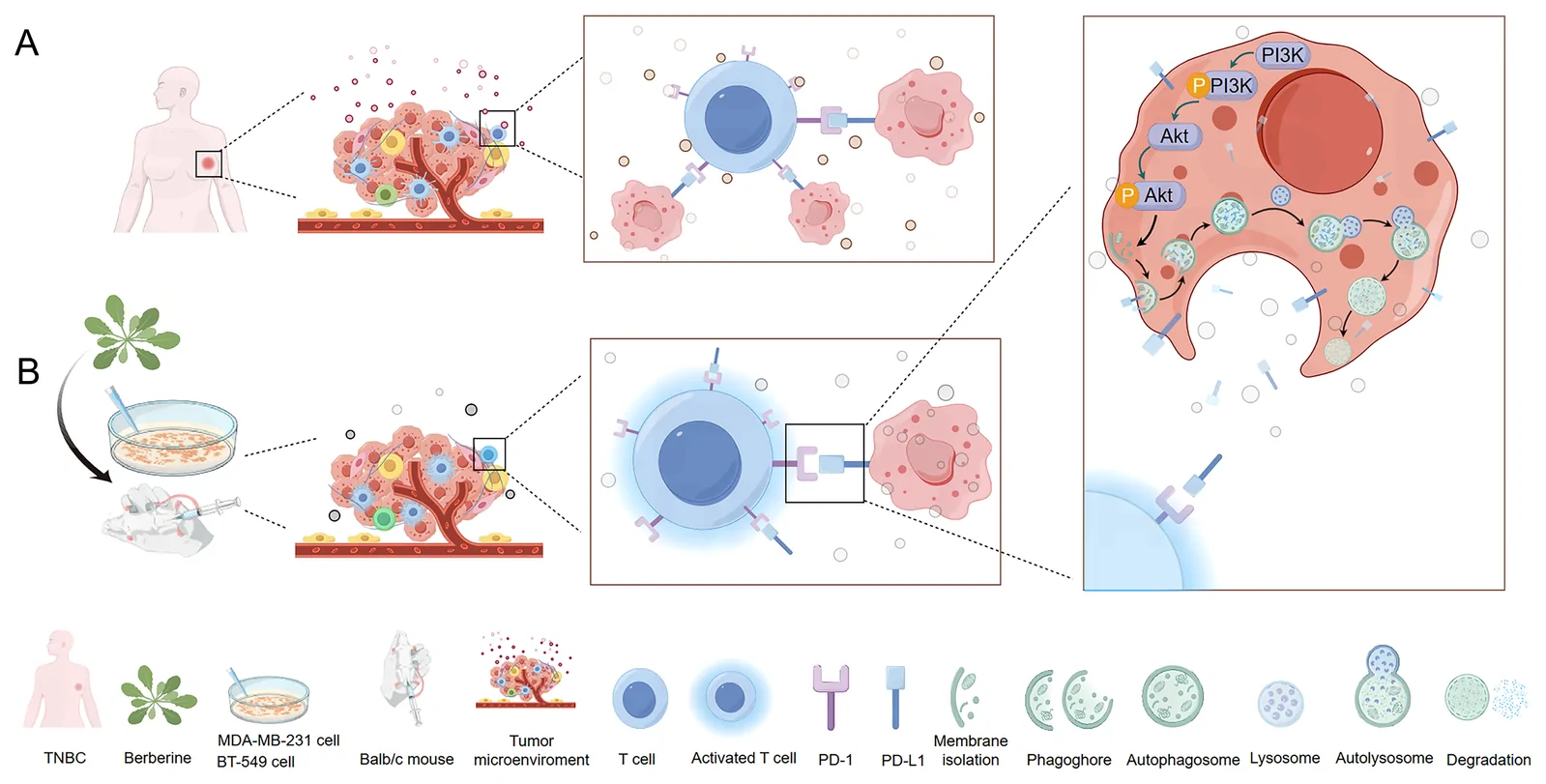
Schematic illustration of the mechanism by which BBR reverses tumor immunosuppression in TNBC via the PI3K-Akt–autophagy–PD-L1 signaling axis. **(A)** Basal immunosuppressive state in TNBC. TNBC cells exhibit high membrane expression of PD-L1, which engages PD-1 on T cells to induce T cell dysfunction and facilitate tumor immune escape within the tumor microenvironment. **(B)** BBR‑driven restoration of anti‑tumor immunity. Validated in TNBC cell lines (MDA-MB-231, BT-549) and BALB/c mouse models, BBR suppresses PI3K-Akt signaling and subsequently promotes the autophagy‑lysosome pathway to mediate PD-L1 degradation. The downregulation of membrane PD-L1 abrogates PD-1/PD-L1 immune checkpoint interaction, thereby restoring T cell activation and potentiating anti‑tumor immune responses.

This study systematically reveals the molecular mechanism by which BBR degrades PD-L1 via the PI3K-Akt pathway-mediated autophagy-lysosomal pathway and enhances the anti-tumor immune response in TNBC (**Figure 10**). The findings establish a novel regulatory axis—BBR-PI3K-Akt-autophagy-PD-L1—that connects a classical metabolic signaling pathway to immune checkpoint regulation, providing both mechanistic insight and a translational rationale for developing small-molecule immunomodulators in TNBC.

A key observation in this study is that BBR reduced PD-L1 protein abundance while qRT-PCR revealed increased PD-L1 mRNA. This inverse pattern is not contradictory but rather reflects the multilayered regulation of PD-L1, which operates at transcriptional, post-transcriptional, translational, and post-translational levels (25). If BBR primarily suppressed PD-L1 transcription, both mRNA and protein would decline in parallel. The observed mRNA rise therefore argues against transcriptional inhibition as the dominant mechanism and instead supports a post-translational mode of action. Cycloheximide chase assays confirmed that BBR markedly shortens PD-L1 half-life, establishing accelerated degradation as the primary event. The increase in PD-L1 mRNA is likely a compensatory transcriptional feedback triggered by rapid protein loss. Under cellular stress, NF-κB can directly induce PD-L1 gene transcription (26). Moreover, PD-L1 mRNA stability can be enhanced through RNA-binding proteins such as TTP (27). These mechanisms may collectively drive the modest mRNA upregulation seen here. Nevertheless, this compensatory rise is insufficient to overcome the robust autophagic-lysosomal degradation enforced by BBR, resulting in a net decrease of PD-L1 protein.

This transcription-translation uncoupling has broader implications for understanding PD-L1 biology. It suggests that PD-L1 protein levels are not reliably predicted by mRNA abundance alone, which is consistent with emerging evidence that post-translational mechanisms—including glycosylation, ubiquitination, palmitoylation, and autophagic turnover—play dominant roles in determining PD-L1 surface expression (39). From a clinical perspective, this finding cautions against using PD-L1 mRNA as a surrogate biomarker for predicting immunotherapy response, and highlights the importance of assessing PD-L1 protein dynamics, particularly degradation kinetics, when evaluating small-molecule checkpoint modulators.

In contrast to a previous report showing that BBR promotes proteasomal PD-L1 degradation via CSN5 inhibition in lung cancer (28), our study demonstrates that in TNBC, BBR acts primarily through the autophagy-lysosome pathway. This conclusion is supported by multiple lines of evidence: the proteasome inhibitor MG132 did not reverse BBR-induced PD-L1 downregulation, whereas lysosomal inhibitors (Baf A1, CQ) and the autophagy inhibitor (3-MA) significantly blocked this effect. Enhanced co-localization of PD-L1 with LC3B/LAMP1 further corroborated autophagic engagement. Notably, Lyso-Tracker Red and acridine orange staining showed that BBR did not affect lysosomal activity or membrane stability, excluding protein degradation caused by non-specific lysosomal damage and indicating that BBR exerts a targeted regulation on PD-L1 through the autophagic machinery rather than through lysosomal disruption.

The pathway specificity of BBR-induced PD-L1 degradation in TNBC versus the proteasome-dependent mechanism in lung cancer merits careful consideration. Several factors may account for this divergence. First, cell type-specific differences in the relative activity of degradation systems: TNBC cells exhibit relatively high basal autophagic flux compared with Lewis lung cancer cells, where the ubiquitin-proteasome system is the dominant protein turnover route (29). Second, the expression level of CSN5—the deubiquitinase targeted by BBR in lung cancer (28)—differs across tumor types; CSN5 is highly expressed in Lewis lung cancer cells, while its expression is relatively low in TNBC cells such as MDA-MB-231, potentially below the threshold for BBR-mediated CSN5 inhibition at the effective concentration (10 μmol/L). Third, recent study on the berberine derivative B68 have shown that C9 alkylation enhances B68’s binding affinity to CSN5 compared with the parent compound BBR, which primarily targets nucleic acids (17), suggesting that structural modification rather than the native scaffold dictates CSN5 engagement. The effective concentration of B68 (20 μmol/L) is higher than that of BBR in this study, further supporting a dose-dependent switch in target engagement.

This study adds to a growing body of literature on autophagy-lysosome–mediated PD-L1 degradation. Hsc70 has been shown to promote PD-L1 degradation via the endosome-lysosome pathway by competitively binding to CMTM6 (8). HIP1R acts as an autophagy receptor that targets PD-L1 to lysosomal degradation through an intrinsic sorting signal (40). Most recently, zosuquidar was found to trigger autophagic PD-L1 degradation by enhancing ABCB1-PD-L1 interaction and causing ER retention followed by SQSTM1/p62-dependent selective autophagy (30), and the TRIM14/USP14 axis was identified as a regulator of PD-L1 autophagic degradation through K63-linked deubiquitination (31). The mechanism described in the present study is distinct from these existing models in several important aspects: (1) Unlike Hsc70, which directly binds to the intracellular domain of PD-L1 and competes with CMTM6 for binding (a receptor-mediated selective degradation), BBR indirectly activates autophagy by inhibiting the PI3K-Akt pathway upstream, representing a pathway-dependent rather than receptor-dependent mechanism. (2) Unlike zosuquidar, which intercepts PD-L1 at the ER-Golgi transport step, BBR-induced autophagic degradation likely targets PD-L1 that has already trafficked to or near the plasma membrane, as evidenced by the significant reduction in membrane PD-L1. (3) BBR does not affect lysosomal activity per se, distinguishing it from the enhanced lysosomal function observed in the Hsc70 study (8); this suggests that BBR achieves PD-L1 degradation by regulating autophagy initiation rather than lysosomal function, which may carry a lower risk of non-specific protein degradation.

At the functional level, BBR not only reduced the total expression of PD-L1 but also specifically decreased its membrane localization. Cellular immunofluorescence showed weakened total fluorescence intensity of PD-L1, while membrane protein extraction WB and flow cytometry further confirmed a significant reduction in membrane PD-L1. This dual regulation has important physiological significance: membrane PD-L1 is the functional molecule mediating tumor immune escape through PD-1 engagement, and its reduction can directly relieve immunosuppression on T cells. Indeed, enhanced cytotoxicity of primary human T cells against BBR-pretreated TNBC cells, supported by supplementary evidence from PBMC-TNBC interaction models, as well as the results of PD-L1/PD-1 binding inhibition experiments consistent with reduced membrane PD-L1, provided direct evidence supporting this functional effect.

It is worth noting that PD-L1 displays spatial heterogeneity—not only on the cell membrane but also in the cytoplasm, nucleus, and exosomes—and each pool carries distinct functional implications (43). Cytoplasmic PD-L1 can translocate to the cell surface to replenish membrane PD-L1, contributing to resistance against anti-PD-L1 antibody therapy (43). Nuclear PD-L1 promotes tumor progression through immune-independent mechanisms (43). By reducing total PD-L1 protein, BBR potentially depletes not only the membrane pool but also the cytoplasmic reservoir that could replenish it, which may confer an advantage over anti-PD-L1 antibodies that only block the membrane-localized fraction. Particularly in the IFN-γ–induced microenvironment model, BBR could still effectively inhibit PD-L1 upregulation, suggesting that it may retain activity in the complex TME—a critical prerequisite for clinical translation, given that IFN-γ is a major driver of adaptive immune resistance in TNBC.

This study is the first to identify the PI3K-Akt pathway as an upstream regulatory node for BBR to activate autophagy-lysosomal degradation of PD-L1. WB assay showed that BBR could downregulate the expression of PI3K, Akt, and their phosphorylated forms, while the PI3K activator 740 Y-P could reverse BBR-induced PD-L1 degradation and changes in autophagy markers. Conversely, treatment with the PI3K inhibitor LY294002 alone could mimic the effect of BBR. These results suggest a cascade regulatory relationship of BBR-PI3K-Akt-autophagy-PD-L1.

It is important to address the potential confounding factor that both 3-MA and LY294002 affect PI3K-related pathways. The PI3K family consists of multiple subunits with distinct functions: Class I PI3K (the primary target of LY294002) regulates Akt signaling and inhibits autophagy through Akt-mTOR activation, while Class III PI3K/Vps34 (the target of 3-MA) regulates autophagy initiation through PI3P production and autophagosome formation. BBR inhibits the Class I PI3K-Akt pathway, relieving Akt-mediated autophagy inhibition. LY294002 (Class I PI3K inhibitor) mimics BBR effects by inhibiting Akt signaling and activating autophagy. Conversely, 3-MA (Class III PI3K inhibitor) blocks autophagy initiation and prevents BBR-induced PD-L1 degradation. Therefore, both inhibitors can block BBR effects, validating autophagy in PD-L1 degradation from two complementary perspectives: upstream signal relief of inhibition (LY294002) and downstream execution blockade (3-MA). The convergence of evidence from both upstream signal modulation and downstream execution blockade strongly supports the regulatory axis involving PI3K, autophagy, and PD-L1.

An apparent paradox deserves discussion. Most studies report that PI3K-Akt activation upregulates PD-L1 (32, 33). This study, however, demonstrates that PI3K-Akt inhibition by BBR degrades PD-L1 through autophagy activation—a mechanism distinct from translational or transcriptional regulation. This contradiction may be resolved by considering the following: (1) Tumor type difference: In non-small cell lung cancer, PI3K-Akt activation mainly mediates PD-L1 translation through the mTOR-S6K/4E-BP1 axis (34); in TNBC, however, the basal activity of PI3K-Akt is higher (35), and its inhibition is more likely to trigger autophagic compensation, creating a mechanistic context where PI3K-Akt blockade redirects PD-L1 fate toward autophagic degradation rather than simply reducing translational output. (2) The dual output of PI3K-Akt signaling: this pathway simultaneously regulates PD-L1 synthesis (via mTOR-mediated translation) and PD-L1 degradation (via autophagy suppression). Under conditions of PI3K-Akt activation, the net effect may be increased PD-L1 because enhanced synthesis outpaces unchanged degradation; under PI3K-Akt inhibition, the net effect is determined by the balance between reduced synthesis and increased autophagic degradation. In TNBC cells, the autophagic degradation pathway appears to dominate, potentially because the autophagic machinery is more responsive to PI3K-Akt modulation in this context. (3) Experimental model difference: The 4T1 model used in this study employs immunocompetent mice, while many other studies using immunodeficient models cannot reflect the feedback regulation of T cells on PD-L1 dynamics. There are reported that anti-PD-L1 antibodies induce autophagy by inhibiting the PI3K-Akt/mTOR pathway (36), which is consistent with the pathway inhibition effect in this study and supports the concept that PI3K-Akt inhibition can function as a physiological trigger for autophagy-mediated PD-L1 turnover. Furthermore, recent work has demonstrated that constitutive mTORC1 activation in PI3K inhibitor–resistant breast cancer suppresses autophagy and creates metabolic vulnerability (44), underscoring the tight link between PI3K-Akt-mTOR signaling and autophagy regulation in breast cancer.

BBR directly competitively binds to the ATP-binding pocket of the PI3K p110α subunit (37), which is mechanistically distinct from PI3K-Akt activation caused by upstream receptor mutations or PTEN loss. This direct enzyme inhibition may produce a more complete and rapid shutdown of PI3K-Akt signaling, triggering a more robust autophagic response than partial pathway modulation. Future studies employing phosphoproteomics could map the downstream signaling nodes that connect PI3K-Akt inhibition to the autophagic machinery with greater precision.

*In vivo* experiments further verified the physiological relevance of this mechanism. In the 4T1 xenograft model, BBR treatment significantly reduced the activity of the PI3K-Akt pathway in tumor tissues, accompanied by PD-L1 downregulation and autophagy activation. Notably, BBR showed no tumor-suppressive effect in the TNBC nude mouse model but was effective in the immunocompetent 4T1 model. This difference strongly suggests that its anti-tumor effect depends on the presence of the immune system, supporting the conclusion that BBR exerts its effect through immune regulation rather than direct cytotoxicity. IHC and flow cytometry results showed that after BBR treatment, the proportion of CD69^+^CD8^+^ activated T cells in the TME increased, granzyme B expression was upregulated, and the number of Tregs decreased—changes that collectively constitute the remodeling of the immune microenvironment, traceable to the relieved immune checkpoint blockade caused by PD-L1 degradation.

This immune-dependent *in vivo* efficacy has important implications for clinical translation. It suggests that BBR’s therapeutic window is defined not by direct tumor cell killing but by its ability to re-engage the host immune system. This mechanistic profile is fundamentally different from conventional chemotherapy and more analogous to immune checkpoint inhibitors. Importantly, comprehensive viability assays and total protein measurements confirmed that the concentration of BBR used for mechanistic studies does not induce significant cytotoxicity or non-specific protein loss in TNBC cells, and exhibits selectivity for cancer cells over normal mammary epithelial cells. The enhanced T cell-mediated killing observed in co-culture experiments was not due to BBR’s direct cytotoxicity, as confirmed by separate viability controls for PBMC. These data collectively support the specificity of BBR’s action on PD-L1 degradation via the PI3K-Akt-autophagy-lysosomal pathway, rather than through non-specific cytotoxic effects.

In addition to PD-L1 regulation, this study found that BBR increased the expression of ICD-associated markers in TNBC cells. Cellular immunofluorescence and WB experiments confirmed that BBR treatment significantly increased the expression of CRT, HMGB1, and HSP70. ICD is a special type of cell death characterized by the release of DAMPs, which can activate dendritic cells and initiate adaptive immune responses when functional ICD is established. This dual-action mode—PD-L1 degradation to relieve immunosuppression combined with ICD-associated marker upregulation to potentially activate immune recognition—is significantly different from traditional PD-L1 antibodies, which only block PD-L1/PD-1 interaction without affecting immunogenicity.

The concept of combining PD-L1 degradation with ICD induction has gained traction in recent literature. Nanoparticle systems that amplify autophagy to evoke ICD combined with anti-PD-1/PD-L1 therapy have shown synergistic antitumor effects (38). In TNBC specifically, co-delivery of doxorubicin (a classical ICD inducer) and BBR using nanoparticles was found to downregulate PD-L1 and eliminate cancer stem cells, improving chemo-immunotherapy responsiveness (39) Compared with these approaches, BBR occupies a unique position: as a single agent, it simultaneously modulates two critical nodes of the cancer-immunity cycle—checkpoint removal (PD-L1 degradation) and antigen visibility (ICD-associated marker expression)—whereas most strategies require drug combinations or engineered delivery systems to achieve the same dual effect. Moreover, BBR at 10 μmol/L showed no significant toxicity to normal mammary epithelial cells, while drugs such as doxorubicin are often accompanied by immune cell damage, suggesting a potentially more favorable therapeutic index.

However, we acknowledge that our current data demonstrate increased expression of ICD-associated markers but do not provide functional validation of bona fide ICD. The upregulation of CRT, HMGB1, and HSP70 represents necessary but not sufficient conditions for ICD. Furthermore, the relationship between BBR-induced autophagy and ICD-associated marker upregulation warrants investigation. Previous studies have shown that autophagy inhibitors (e.g., 3-MA) can attenuate ICD effects (40) suggesting that the autophagy-ICD marker-PD-L1 degradation axis may constitute a novel regulatory network in which autophagy simultaneously drives both PD-L1 degradation and DAMP exposure. This represents a limitation of the current study and an important direction for future research. A limitation of the present study is that the differential adherence method was used for lymphocyte enrichment rather than MACS-based negative selection, which would have provided higher T cell purity. Although the subsequent CD3/CD28 activation and IL-2 expansion functionally enriched the T cell population, the possibility of residual contamination with non-T mononuclear cells cannot be entirely excluded.

From the perspective of clinical translation, the safety profile of BBR provides an important foundation for its application. In the 4T1 model, there were no abnormalities in body weight and HE staining of major organs in the BBR treatment group, which is consistent with the low toxicity to normal mammary epithelial MCF-10A cells observed in previous studies. The observation that BBR increased metabolic activity in normal MCF-10A cells but inhibited growth in TNBC cells at higher concentrations aligns with previous studies demonstrating the biphasic (hormetic) effects of BBR (18, 41, 42). At lower concentrations, BBR activates AMPK and stimulates mitochondrial biogenesis, enhancing cellular metabolism (43, 44). This metabolic stimulation is particularly pronounced in normal cells, potentially reflecting their intact stress response pathways (45). In contrast, cancer cells, which often exhibit metabolic dysregulation, are more susceptible to the cytotoxic effects of higher BBR concentrations that disrupt mitochondrial membrane potential and induce apoptosis (46, 47). The differential response creates a therapeutic window where normal cells may benefit from BBR-induced metabolic enhancement, while cancer cells are selectively targeted at higher doses.

It should be noted, however, that the hormetic effect of BBR also raises caution. It is reported that low-dose BBR (1.25–5 μM) can promote cancer cell proliferation and attenuate the anticancer activity of chemotherapeutic agents through activation of MAPK/ERK1/2 and PI3K/AKT signaling pathways. Subsequent work confirmed that low-dose BBR induces autophagy and antioxidant responses that antagonize chemotherapy in breast cancer cells (48). These findings underscore the importance of dose optimization in clinical translation: the immunomodulatory dose (10 μmol/L in this study) must be carefully distinguished from doses that may produce hormetic pro-tumor effects. Nanocarrier-based delivery systems, such as the recently described BBR-loaded thermosensitive hydrogel nanoparticles that achieved sustained release and synergized with anti-PD-L1 therapy in TNBC (49), may help maintain BBR at the effective concentration range at the tumor site while avoiding systemic exposure to low, potentially stimulatory doses.

The discovery of BBR as a PD-L1 degrader places it within the rapidly evolving landscape of small-molecule PD-L1-targeting strategies. PROteolysis-Targeting Chimeras (PROTACs) have emerged as a powerful platform for PD-L1 degradation. Several PROTAC molecules—such as compound P22 (lysosome-dependent degradation) (50), compound 21a (proteasome-dependent degradation) (51), and the PD-L1/SHP2 dual PROTACs (52)—have demonstrated effective PD-L1 degradation and antitumor activity in preclinical models. More recently, PD-L1-specific peptide-drug conjugates have been developed as new degraders with improved selectivity (53) Compared with these engineered molecules, BBR has several distinctive features: (1) it is a naturally occurring compound with a well-established safety record and extensive pharmacokinetic data from clinical use in metabolic diseases; (2) it degrades PD-L1 via the autophagy-lysosome pathway rather than the proteasome, which may circumvent resistance mechanisms associated with proteasome-dependent degradation, such as compensatory upregulation of deubiquitinases; (3) its multi-target nature—including PI3K inhibition, autophagy activation, and ICD-associated marker upregulation—may produce broader immunomodulatory effects than single-target PROTACs, though this also introduces complexity in mechanistic dissection and dose optimization.

However, BBR also has well-known limitations compared with designed degraders, most notably its poor oral bioavailability and low aqueous solubility. These pharmacokinetic challenges must be addressed before clinical translation, potentially through nanocarrier formulations or structural optimization (as exemplified by the BBR derivative B68).

This study has several important limitations that should be acknowledged. First, the lack of genetic validation (e.g., ATG5/ATG7 siRNA knockdown or knockout) to directly confirm that PD-L1 degradation is autophagy-dependent represents a significant gap. Although the consistency of multi-faceted pharmacological evidence—including three autophagy/lysosome inhibitors with distinct mechanisms, PI3K pathway modulators, LC3-II turnover quantification, and immunofluorescence co-localization—provides robust support for the proposed mechanism, genetic approaches would provide definitive causality. Future studies will supplement ATG5/ATG7 knockdown experiments to validate this autophagy dependence.

Second, the conclusion regarding proteasome independence is based solely on pharmacological inhibition with MG132. While MG132 failed to rescue PD-L1 levels, this observation does not formally rule out the participation of ubiquitination in BBR-mediated PD-L1 degradation. K63-linked ubiquitin chains can function as recognition signals for autophagic cargo receptors such as p62/SQSTM1 (54), and the role of specific ubiquitin linkages in BBR-induced PD-L1 degradation remains unexplored. Future studies employing ubiquitination immunoprecipitation assays and linkage-specific ubiquitin antibodies would provide direct evidence to clarify whether ubiquitin modifications participate in the autophagic targeting of PD-L1.

Third, the downstream key molecules of the PI3K-Akt pathway regulating the autophagy-lysosomal pathway were not explored in depth. The molecular cascade connecting PI3K-Akt inhibition to autophagy initiation—whether through mTORC1, ULK1 complex, Beclin-1, or other intermediaries—remains to be delineated. Phosphoproteomic and interactomic analyses would help identify the specific regulatory nodes.

Fourth, the human sample component of this study has significant limitations. The serum and tissue cohorts were small and heterogeneous, comprising breast cancer patients of mixed subtypes rather than exclusively TNBC. Therefore, these data should be viewed as exploratory and hypothesis-generating; they provide initial support for the relevance of PD-L1 in breast cancer but do not permit definitive conclusions about TNBC specifically. Future studies with larger, well-annotated, TNBC-specific cohorts are necessary.

Fifth, the functional validation of ICD remains incomplete, as discussed above. Whether BBR induces bona fide ICD in TNBC—and whether autophagy is required for this effect—requires vaccination/rechallenge experiments, dendritic cell cross-presentation assays, and CRT surface exposure measurement.

Finally, the translational potential of BBR is constrained by its poor oral bioavailability, and the effective concentration *in vitro* (10 μmol/L) may not be readily achievable at the tumor site through systemic administration. Future studies should focus on: (1) optimization of drug delivery using nanocarriers (e.g., exosomes, thermosensitive hydrogels) to improve tumor enrichment and reduce PI3K inhibitory toxicity to normal tissues; (2) biomarker screening—TNBC patients with PD-L1/LC3B double positivity may be more likely to benefit from BBR treatment, and this prediction model needs to be validated in clinical trials; (3) combination strategies with existing immunotherapies (anti-PD-1/PD-L1 antibodies) or chemotherapy to assess additive or synergistic effects.

## 5 Conclusion

In summary, through multi-level experiments, this study provides strong evidence that BBR activates the autophagy-lysosomal pathway by inhibiting the PI3K-Akt pathway, specifically degrades PD-L1 protein, and simultaneously increases the expression of ICD-associated markers, ultimately relieving tumor immunosuppression and suggesting potential activation of adaptive immune responses. The elucidation of this mechanism reveals a new biological function of BBR and establishes a novel regulatory axis of “PI3K-Akt-autophagy-PD-L1”. This axis is mechanistically distinct from previously described PD-L1 degradation pathways—whether proteasome-dependent (CSN5-mediated) (55) or receptor-mediated selective autophagy [Hsc70/CMTM6 (8), HIP1R (56), ABCB1/zosuquidar (30)]—and uniquely demonstrates that pathway-level intervention (PI3K-Akt inhibition) can redirect PD-L1 fate toward autophagic destruction. For TNBC, a malignant tumor lacking effective targets and with a low response rate to immunotherapy, the discovery of BBR provides a theoretical basis for the development of novel small-molecule immunomodulators and highlights the potential of targeting intracellular signaling-degradation circuitry as a complement to antibody-based checkpoint blockade.

## Data Availability

The original contributions presented in the study are included in the article/**Supplementary Material**. Further inquiries can be directed to the corresponding author.

## Supplementary material

Supplementary Figure 1 Determination of working concentrations of pharmacological reagents in TNBC cells by CCK-8 assay. Cell viability of MDA-MB-231 **(A–H)** and BT-549 **(I–P)** cells following treatment with increasing concentrations of the indicated reagents for 24 h. **(A, I)** 3-MA; **(B, J)** rapamycin (Rapa); **(C, K)** bafilomycin A1 (BafA1); **(D, L)** chloroquine (CQ); **(E, M)** cycloheximide (CHX); **(F, N)** MG132; **(G, O)** 740 Y-P; **(H, P)** LY294002. Data are presented as mean ± SEM (n=3 independent experiments). ns, not significant; *P<0.05, **P<0.01, ***P<0.001, ****P<0.0001 vs. the control group (0 concentration). Data are presented as mean ± SEM. ns, not significant. **P* < 0.05, ***P* < 0.01, ****P* < 0.001, *****P* < 0.0001 vs. the corresponding control group.

Supplementary Figure 2 BBR regulates cell viability of distinct cell lines in dose- and time-dependent patterns. Cell viability of MDA-MB-231, BT-549, 4T1 and MCF-10A cells was evaluated by CCK-8 assay after incubation with gradient BBR concentrations for 12 h **(A–D)**, 24 h **(E–H)** and 48 h **(I–L)**. Viability data were normalized to untreated control cells. BBR exerted a biphasic regulatory effect, promoting cell activity at low doses while suppressing cell viability at high doses, which conforms to the typical hormetic response of plant-derived compounds. Data are presented as mean ± SEM. ns, not significant. **P* < 0.05, ***P* < 0.01, ****P* < 0.001, *****P* < 0.0001 vs. the corresponding control group.

Supplementary Figure 3 Supplementary validation assays of BBR effects in TNBC cells, peripheral blood mononuclear cells and clinical specimens. **(A)** Serum soluble PD-L1 levels from healthy donors and breast cancer patients detected by ELISA. **(B)** PD-L1 expression in paired tumor and adjacent normal breast tissues examined via WB, with GAPDH as internal control. **(C, D)** Total protein content of two TNBC cell lines after 24-hour treatment with gradient BBR concentrations determined by BCA assay. **(E–G)** Cell apoptosis detected by flow cytometry in BBR-exposed TNBC cells. Representative plots and apoptotic ratio statistics are displayed. **(H–K)** Flow cytometric detection of surface CD44 expression in TNBC cells with or without BBR intervention. **(L, M)** Cell viability of TNBC cells co-cultured with PBMCs at varying effector-target ratios measured by CCK-8 assay. **(N, O)** PBMC viability after exposure to different BBR doses for 12 h and 24 h. Data are presented as mean ± SEM. ns, not significant. **P* < 0.05, ***P* < 0.01, ****P* < 0.001, *****P* < 0.0001 vs. the corresponding control group.

Supplementary Figure 4 Functional verification of BBR anti-tumor efficacy and biosafety *in vitro* and *in vivo*. **(A, B)** CCK-8 assay detects viability of IFN-γ-stimulated TNBC cells co-cultured with PBMCs. **(C)** Crystal violet staining shows survival status of TNBC cells under different co-culture and treatment conditions. **(D)** Representative macroscopic images of MDA-MB-231 xenograft tumors from nude mice. **(E–G)** Dynamic body weight, tumor volume and final tumor weight of mice in each group. **(H)** H&E staining of major visceral organs observed at two magnifications (scale bars: 200 μm, 50 μm). Data are presented as mean ± SEM. ns, not significant. **P* < 0.05, ***P* < 0.01, ****P* < 0.001, *****P* < 0.0001 vs. the corresponding control group.

Supplementary Figure 5 BBR induces PD-L1 degradation through autophagy-lysosome fusion modulated by autophagy initiation and lysosomal formation. **(A, B)** Representative immunofluorescence co-localization of PD-L1 and LC3B in MDA-MB-231 and BT-549 cells under different treatments. Relative fluorescence intensity was quantified to reflect changes in PD-L1 and LC3B abundance. **(C, D)** Co-staining of lysosomal marker LAMP1 and PD-L1 in two TNBC cell lines. Quantitative results illustrate the regulatory effects of BBR, BafA1 and 3-MA on LAMP1 expression and PD-L1 stability. **(E, F)** Double immunofluorescence staining of LC3B and LAMP1. Quantitative analysis reveals the impacts of different treatments on autophagosome-lysosome fusion process. Scale bar = 20 μm (n=3 independent experiments for each condition). Data are presented as mean ± SEM. ns, not significant. **P* < 0.05, ***P* < 0.01, ****P* < 0.001, *****P* < 0.0001 vs. the corresponding control group.

## Glossary

TNBC: Triple-Negative Breast Cancer
BBR: Berberine
PD-L1: Programmed Death-Ligand 1
PD-1: Programmed Cell Death Protein 1
ICB: Immune Checkpoint Blockade
TME: Tumor Microenvironment
TILs: Tumor-Infiltrating Lymphocytes
IFN-γ: Interferon-γ
ICD: Immunogenic Cell Death
PI3K: Phosphatidylinositol 3-Kinase
Akt: Protein Kinase B
p-PI3K: Phosphorylated Phosphatidylinositol 3-Kinase
p-Akt: Phosphorylated Protein Kinase B
WB: Western Blot
ELISA: Enzyme-Linked Immunosorbent Assay
CHX: Cycloheximide
Baf A1: Bafilomycin A1
CQ: Chloroquine
3-MA: 3-Methyladenine
MG132: Carbobenzoxy-Leucyl-Leucyl-Leucinal
CRT: Calreticulin
HMGB1: High-Mobility Group Box 1
HSP70: Heat Shock Protein 70
DAMPs: Damage-Associated Molecular Patterns
LAMP1: Lysosome-Associated Membrane Protein 1
FACS: Flow Cytometry
MFI: Mean Fluorescence Intensity
IHC: Immunohistochemistry
BSA: Bovine Serum Albumin
PBS: Phosphate-Buffered Saline
DMSO: Dimethyl Sulfoxide
SDS-PAGE: Sodium Dodecyl Sulfate-Polyacrylamide Gel Electrophoresis
PVDF: Polyvinylidene Difluoride
TBST: Tris-Buffered Saline with Tween 20
ECL: Enhanced Chemiluminescence
GAPDH: Glyceraldehyde-3-Phosphate Dehydrogenase
BCA: Bicinchoninic Acid
DAPI: 4’,6-Diamidino-2-Phenylindole
LDH: Lactate Dehydrogenase
ANOVA: Analysis of Variance
Tregs: Regulatory T Cells
CSN5: COP9 Signalosome Subunit 5
BMI1: B-Cell-Specific Moloney Murine Leukemia Virus Integration Site 1
EGFR: Epidermal Growth Factor Receptor
PTEN: Phosphatase and Tensin Homolog
mTOR: Mammalian Target of Rapamycin
RKO: Human Colorectal Carcinoma Cell Line
HCT116: Human Colorectal Carcinoma Cell Line
MCF-10A: Human Normal Mammary Epithelial Cell Line.

## References

[1] Huang Z Peng Q Mao L Ouyang W Xiong Y Tan Y . Neoadjuvant strategies for triple negative breast cancer: Current evidence and future perspectives. MedComm - Future Med. (2025) 4:e70013. doi: 10.1002/mef2.70013 41531421

[2] Topalian SL Forde PM Emens LA Yarchoan M Smith KN Pardoll DM . Neoadjuvant immune checkpoint blockade: A window of opportunity to advance cancer immunotherapy. Cancer Cell. (2023) 41:1551–66. doi: 10.1016/j.ccell.2023.07.011 37595586 PMC10548441

[3] Yang Z Liu X Zhu J Chai Y Cong B Li B . Inhibiting intracellular CD28 in cancer cells enhances antitumor immunity and overcomes anti-PD-1 resistance via targeting PD-L1. Cancer Cell. (2025) 43:86–102.e110. doi: 10.1016/j.ccell.2024.11.008 39672166

[4] Lin Q Liu T Wang X Hou G Xiang Z Zhang W . Long noncoding RNA HITT coordinates with RGS2 to inhibit PD-L1 translation in T cell immunity. J Clin Invest. (2023) 133(11):e162951. doi: 10.1172/jci162951 37014700 PMC10231998

[5] Hu L Sun C Yuan K Yang P . Expression, regulation, and function of PD-L1 on non-tumor cells in the tumor microenvironment. Drug Discov Today. (2024) 29:104181. doi: 10.1016/j.drudis.2024.104181 39278561

[6] Zhu Z Yang J Montefusco S Xia S Ou J Tong H . Proteotoxic stress triggers TFEB- and TFE3-mediated autophagy and lysosomal biogenesis via non-canonical MTORC1 inactivation. Autophagy. (2026) 22(4):726–43. doi: 10.1080/15548627.2025.2608973 41450115 PMC13020891

[7] Zhang Y Liu X Klionsky DJ Lu B Zhong Q . Manipulating autophagic degradation in human diseases: from mechanisms to interventions. Life Med. (2022) 1:120–48. doi: 10.1093/lifemedi/lnac043 39871921 PMC11749641

[8] Xu X Xie T Zhou M Sun Y Wang F Tian Y . Hsc70 promotes anti-tumor immunity by targeting PD-L1 for lysosomal degradation. Nat Commun. (2024) 15:4237. doi: 10.1038/s41467-024-48597-3 38762492 PMC11102475

[9] Jing W Jie C Chun H Cheng H . PI3K/AKT regulates the expression of PD-L1 via Nrf2 pathway in non-small cell lung cancer. China Oncol. (2020) 30:419–27. doi: 10.19401/j.cnki.1007-3639.2020.06.003

[10] Quan Z Yang Y Zheng H Zhan Y Luo J Ning Y . Clinical implications of the interaction between PD-1/PD-L1 and PI3K/AKT/mTOR pathway in progression and treatment of non-small cell lung cancer. J Cancer. (2022) 13:3434–43. doi: 10.7150/jca.77619 36313041 PMC9608206

[11] Mei W Wei M Tang C Li W Ye B Xin S . BCAT2 binding to PCBP1 regulates the PI3K/AKT signaling pathway to inhibit autophagy-related apoptosis and ferroptosis in prostate cancer. Cell Death Dis. (2025) 16:337. doi: 10.1038/s41419-025-07559-3 40274762 PMC12022009

[12] Fan KQ Zhang L Song F Zhang YH Chen T Cheng X . Pharmacological properties and therapeutic potential of berberine: a comprehensive review. Front Pharmacol. (2025) 16:1604071. doi: 10.3389/fphar.2025.1604071 40894216 PMC12391081

[13] Zhou W Asif A Situ C Wang J Hao H . Multiple target and regulatory pathways of berberine. Phytomedicine. (2025) 146:157030. doi: 10.1016/j.phymed.2025.157030 40763599

[14] Xiong RG Huang SY Wu SX Zhou DD Yang ZJ Saimaiti A . Anticancer effects and mechanisms of berberine from medicinal herbs: An update review. Molecules. (2022) 27(14):4523. doi: 10.3390/molecules27144523 35889396 PMC9316001

[15] Chen G Zhang C Zou J Zhou Z Zhang J Yan Y . Coptidis rhizoma and berberine as anti-cancer drugs: A 10-year updates and future perspectives. Pharmacol Res. (2025) 216:107742. doi: 10.1016/j.phrs.2025.107742 40258505

[16] Cheng H Li X Wang Y Deng W Sun G Zhang D . Berberine pharmacological properties and therapeutic potential across cancer, digestive, metabolic, cardiovascular, and neurological diseases: an update review. Excli J. (2025) 24:1225–61. doi: 10.3389/fphar.2025.1604071 PMC12511532 41078566

[17] Hu H Wang Q Yu D Tao X Guo M Tian S . Berberine derivative B68 promotes tumor immune clearance by dual-targeting BMI1 for senescence induction and CSN5 for PD-L1 degradation. Adv Sci (Weinh). (2025) 12:e2413122. doi: 10.1002/advs.202413122 39721027 PMC11831439

[18] Deng J Kuang W Chen W Zhou E Wu Y Yang Z . Intratumoral immune-microbial crosstalk shaped by tumor cell-derived extracellular vesicles encapsulating berberine boosts lung cancer immunotherapy. Acta Pharm Sin B. (2026) 16:1090–115. doi: 10.1016/j.apsb.2025.12.014 41685138 PMC12892072

[19] Lv J Cai J Yuan K Wang J Xu Y Huang X . Immunogenic cell death inducers: Current advances and future perspectives. Coord Chem Rev. (2025) 542:216846. doi: 10.1016/j.ccr.2025.216846

[20] Hassan SN Ahmad F . Considering dimethyl sulfoxide solvent toxicity to mammalian cells and its biological effects. Exp Oncol. (2024) 46:174–8. doi: 10.15407/exp-oncology.2024.02.174 39396166

[21] Nguyen Sinh Truong Nguyen Huyen Thi-Lam Truong Kiet Dinh . Comparative cytotoxic effects of methanol, ethanol and DMSO on human cancer cell lines. Biomed Res Ther. (2020) 7(7):3855–9. doi: 10.15419/bmrat.v7i7.614

[22] Foulke JG Chen L Chang H McManus CE Tian F Gu Z . Optimizing ex vivo CAR-T cell-mediated cytotoxicity assay through multimodality imaging. Cancers (Basel). (2024) 16(14):2497. doi: 10.3390/cancers16142497 39061136 PMC11274748

[23] Gou Y Yuan X Fan D Yang Y Yuan H . Novel tri-specific immuno-modulatory antibody combined with HDACi to potentiate T cell activation and anti-tumor efficacy. J Cancer. (2025) 16:3907–16. doi: 10.7150/jca.108922 41049003 PMC12490976

[24] Qiu S Han QY Zhao X Li WJ Li XQ . A preliminary study on the therapeutic role of γδT cells in triple-negative breast cancer. Kaohsiung J Med Sci. (2025) 41:e70029. doi: 10.1002/kjm2.70029 40289760 PMC12407383

[25] Yamaguchi H Hsu JM Yang WH Hung MC . Mechanisms regulating PD-L1 expression in cancers and associated opportunities for novel small-molecule therapeutics. Nat Rev Clin Oncol. (2022) 19:287–305. doi: 10.1038/s41571-022-00601-9 35132224

[26] Antonangeli F Natalini A Garassino MC Sica A Santoni A Di Rosa F . Regulation of PD-L1 expression by NF-κB in cancer. Front Immunol. (2020) 11:584626. doi: 10.3389/fimmu.2020.584626 33324403 PMC7724774

[27] Coelho MA de Carné Trécesson S Rana S Zecchin D Moore C Molina-Arcas M . Oncogenic RAS signaling promotes tumor immunoresistance by stabilizing PD-L1 mRNA. Immunity. (2017) 47:1083–1099.e1086. doi: 10.1016/j.immuni.2017.11.016 29246442 PMC5746170

[28] Liu Y Liu X Zhang N Yin M Dong J Zeng Q . Berberine diminishes cancer cell PD-L1 expression and facilitates antitumor immunity via inhibiting the deubiquitination activity of CSN5. Acta Pharm Sin B. (2020) 10:2299–312. doi: 10.1016/j.apsb.2020.06.014 33354502 PMC7745128

[29] Rao K Zhang X Luo Y Xia Q Jin Y He J . Lactylation orchestrates ubiquitin-independent degradation of cGAS and promotes tumor growth. Cell Rep. (2025) 44:115441. doi: 10.1016/j.celrep.2025.115441 40106438

[30] Ding L Guo H Zhang J Zheng M Zhang W Wang L . Zosuquidar promotes antitumor immunity by inducing autophagic degradation of PD-L1. Adv Sci (Weinh). (2024) 11:e2400340. doi: 10.1002/advs.202400340 39229920 PMC11538701

[31] Liu D Li M Zhao Z Zhou L Zhi F Guo Z . Targeting the TRIM14/USP14 axis enhances immunotherapy efficacy by inducing autophagic degradation of PD-L1. Cancer Res. (2024) 84:2806–19. doi: 10.1158/0008-5472.can-23-3971 38924473

[32] Gao Y Feng Y Liu S Zhang Y Wang J Qin T . Immune-independent acquired resistance to PD-L1 antibody initiated by PD-L1 upregulation via PI3K/AKT signaling can be reversed by anlotinib. Cancer Med. (2023) 12:15337–49. doi: 10.1002/cam4.6195 PMC10417303 37350549

[33] Chen S Zhang P Zhu G Wang B Cai J Song L . Targeting GSDME-mediated macrophage polarization for enhanced antitumor immunity in hepatocellular carcinoma. Cell Mol Immunol. (2024) 21:1505–21. doi: 10.1038/s41423-024-01231-0 39496854 PMC11607431

[34] Qiang M Chen Z Liu H Dong J Gong K Zhang X . Targeting the PI3K/AKT/mTOR pathway in lung cancer: mechanisms and therapeutic targeting. (2025) 16:1516583. doi: 10.3389/fphar.2025.1516583 PMC11877449 40041495

[35] Tang K Bai S Zhou Q Liu S Yin L Tong Y . MicroRNA-195-5p targets MYB to regulate proliferation and Malignant metastasis in triple-negative breast cancer via PI3K/AKT/mTOR signaling. Breast J. (2025) 2025:7303173. doi: 10.1155/tbj/7303173 41141380 PMC12549211

[36] Zhang G Chen Y Huang X Liang T . Cancer immunotherapeutic challenges from autophagy-immune checkpoint reciprocal regulation. Trends Cancer. (2025) 11:169–84. doi: 10.1016/j.trecan.2024.11.001 39706727

[37] Conduit SE Vasan N Brown JR Perry MWD Vanhaesebroeck B . A renaissance in targeting the PI3K/AKT/mTOR pathway. Nat Rev Drug Discov. (2026) 25:469–93. doi: 10.1038/s41573-026-01388-5 41912890

[38] Yu J He X Wang Z Wang Y Liu S Li X . Combining PD-L1 inhibitors with immunogenic cell death triggered by chemo-photothermal therapy via a thermosensitive liposome system to stimulate tumor-specific immunological response. Nanoscale. (2021) 13:12966–78. doi: 10.1039/d1nr03288g 34477780

[39] Cheng X Cai H Li X Zhang Y Li S Li Y . Reversing adenosine-mediated immunosuppression in triple-negative breast cancer by synergistic chemo-immunotherapy via stimuli-responsive nanomedicines. EBioMedicine. (2026) 123:106059. doi: 10.1016/j.ebiom.2025.106059 41352123 PMC12721312

[40] Li C Guan XM Wang RY Xie YS Zhou H Ni WJ . Berberine mitigates high glucose-induced podocyte apoptosis by modulating autophagy via the mTOR/P70S6K/4EBP1 pathway. Life Sci. (2020) 243:117277. doi: 10.1016/j.lfs.2020.117277 31926252

[41] Han B Wang K Tu Y Tan L He C . Low-dose berberine attenuates the anti-breast cancer activity of chemotherapeutic agents via induction of autophagy and antioxidation. Dose Response. (2020) 18:1559325820939751. doi: 10.1177/1559325820939751 33100936 PMC7549173

[42] Yang X Liang S Huang M Yue S Jiang D Yan D . Berberine protects pancreatic β-cells from IL-1β damage through hormetic mechanisms via P53-mediated apoptosis pathways. Endocrinology. (2025) 166(9):bqaf105. doi: 10.1210/endocr/bqaf105 40488312

[43] Lantier L Williams AS Williams IM Yang KK Bracy DP Goelzer M . SIRT3 is crucial for maintaining skeletal muscle insulin action and protects against severe insulin resistance in high-fat-fed mice. Diabetes. (2015) 64:3081–92. doi: 10.2337/db14-1810 25948682 PMC4542443

[44] Yadawa AK Srivastava P Singh A Kumar R Arya JK Rizvi SI . Berberine attenuates brain aging via stabilizing redox homeostasis and inflammation in an accelerated senescence model of Wistar rats. Metab Brain Dis. (2024) 39:649–59. doi: 10.1007/s11011-024-01350-7 38727934

[45] Lin X Zhang J Chu Y Nie Q Zhang J . Berberine prevents NAFLD and HCC by modulating metabolic disorders. Pharmacol Ther. (2024) 254:108593. doi: 10.1016/j.pharmthera.2024.108593 38301771

[46] Sun Q Tu K Xu Q Yan L Yang S Wang J . Berberine suppresses colorectal cancer progression by inducing ferroptosis-mediated energy metabolism disorders. J Adv Res. (2025) 61(2):207–14. doi: 10.1016/j.jare.2025.10.025 41139018 PMC13316491

[47] Sun Y Zhou Q Chen F Gao X Yang L Jin X . Berberine inhibits breast carcinoma proliferation and metastasis under hypoxic microenvironment involving gut microbiota and endogenous metabolites. Pharmacol Res. (2023) 193:106817. doi: 10.1016/j.phrs.2023.106817 37315824

[48] Zheng X Zhao Y Jia Y Shao D Zhang F Sun M . Biomimetic co-assembled nanodrug of doxorubicin and berberine suppresses chemotherapy-exacerbated breast cancer metastasis. Biomaterials. (2021) 271:120716. doi: 10.1016/j.biomaterials.2021.120716 33621894

[49] Jin W Xu S Zhang H Li Y Shi M Ma N . A CD36-targeting thermosensitive berberine nanogel blocks tumor lipid hijacking and potentiates anti-PD-L1 immunotherapy in triple-negative breast cancer. Mol Pharm. (2026) 23:958–74. doi: 10.1021/acs.molpharmaceut.5c01378 41567101

[50] Cheng B Ren Y Cao H Chen J . Discovery of novel resorcinol diphenyl ether-based PROTAC-like molecules as dual inhibitors and degraders of PD-L1. Eur J Med Chem. (2020) 199:112377. doi: 10.1016/j.ejmech.2020.112377 32388281

[51] Wang Y Zhou Y Cao S Sun Y Dong Z Li C . *In vitro* and *in vivo* degradation of programmed cell death ligand 1 (PD-L1) by a proteolysis targeting chimera (PROTAC). Bioorg Chem. (2021) 111:104833. doi: 10.1016/j.bioorg.2021.104833 33839580

[52] Liu Y Liang J Zheng M Song H Chen L Li H . PD-L1/SHP2 dual PROTACs inhibit melanoma by enhancing T-cell killing activity. Chin Chem Lett. (2025) 36:110317. doi: 10.1016/j.cclet.2024.110317 38826717

[53] Chen D Xu R Ye X Xiao Y Wang M Li W . Design of a bispecific peptide-nanozyme conjugate for cancer immunotherapy. Cell Rep Med. (2026) 7:102568. doi: 10.1016/j.xcrm.2025.102568 41570818 PMC12923962

[54] Sun D Wu R Zheng J Li P Yu L . Polyubiquitin chain-induced p62 phase separation drives autophagic cargo segregation. Cell Res. (2018) 28:405–15. doi: 10.1038/s41422-018-0017-7 29507397 PMC5939046

[55] Wang Q Guo M Song Y Cui K Hu H Xue X . Chemical space navigation of nitidine leads to the discovery of a novel PD-L1 degradation agent by targeting CSN5 for enhanced antitumor immunity. J Med Chem. (2026) 69:8237–54. doi: 10.1021/acs.jmedchem.5c03636 41824767

[56] Wang H Yao H Li C Shi H Lan J Li Z . HIP1R targets PD-L1 to lysosomal degradation to alter T cell–mediated cytotoxicity. Nat Chem Biol. (2019) 15:42–50. doi: 10.1038/s41589-018-0161-x 30397328

