# Supplementary material for: Berberine induces PD-L1 degradation via the autophagy-lysosome pathway through the PI3K-Akt pathway and enhances immunogenic cell death in triple-negative breast cancer
Source: Front Immunol. 2026 Aug 7;17:1797840. doi: 10.3389/fimmu.2026.1797840 (PMC13493310; doi:10.3389/fimmu.2026.1797840)
Supplement: Supplementary file 1 [file DataSheet1.docx]

**Supplementary Figure S1: Determination of working concentrations of pharmacological reagents in TNBC cells by CCK-8 assay.**

Cell viability of MDA-MB-231 (A-H) and BT-549 (I-P) cells following treatment with increasing concentrations of the indicated reagents for 24 h. **(A, I)** 3-MA; **(B, J)** rapamycin (Rapa); **(C, K)** bafilomycin A1 (BafA1); **(D, L)** chloroquine (CQ); **(E, M)** cycloheximide (CHX); **(F, N)** MG132; **(G, O)** 740 Y-P; **(H, P)** LY294002. Data are presented as mean ± SEM (n=3 independent experiments). ns, not significant; *P<0.05, **P<0.01, ***P<0.001, ****P<0.0001 vs. the control group (0 concentration). Data are presented as mean ± SEM. ns, not significant. **P*<0.05, ***P*<0.01, ****P*<0.001, *****P*<0.0001 vs. the corresponding control group.

**Supplementary Figure S2: BBR regulates cell viability of distinct cell lines in dose- and time-dependent patterns.**

Cell viability of MDA-MB-231, BT-549, 4T1 and MCF-10A cells was evaluated by CCK-8 assay after incubation with gradient BBR concentrations for 12 h **(A-D)**, 24 h **(E-H)** and 48 h **(I-L)**. Viability data were normalized to untreated control cells. BBR exerted a biphasic regulatory effect, promoting cell activity at low doses while suppressing cell viability at high doses, which conforms to the typical hormetic response of plant-derived compounds. Data are presented as mean ± SEM. ns, not significant. **P*<0.05, ***P*<0.01, ****P*<0.001, *****P*<0.0001 vs. the corresponding control group.

**Supplementary Figure S3: Supplementary validation assays of BBR effects in TNBC cells, peripheral blood mononuclear cells and clinical specimens.**

**(A)** Serum soluble PD-L1 levels from healthy donors and breast cancer patients detected by ELISA. **(B)** PD-L1 expression in paired tumor and adjacent normal breast tissues examined via WB, with GAPDH as internal control. **(C, D)** Total protein content of two TNBC cell lines after 24-hour treatment with gradient BBR concentrations determined by BCA assay. **(E-G)** Cell apoptosis detected by flow cytometry in BBR-exposed TNBC cells. Representative plots and apoptotic ratio statistics are displayed. **(H–K)** Flow cytometric detection of surface CD44 expression in TNBC cells with or without BBR intervention. **(L, M)** Cell viability of TNBC cells co-cultured with PBMCs at varying effector-target ratios measured by CCK-8 assay. **(N, O)** PBMC viability after exposure to different BBR doses for 12 h and 24 h. Data are presented as mean ± SEM. ns, not significant. **P*<0.05, ***P*<0.01, ****P*<0.001, *****P*<0.0001 vs. the corresponding control group.

**Supplementary Figure S4: Functional verification of BBR anti-tumor efficacy and biosafety in vitro and in vivo.**

**(A, B)** CCK-8 assay detects viability of IFN-γ-stimulated TNBC cells co-cultured with PBMCs. **(C)** Crystal violet staining shows survival status of TNBC cells under different co-culture and treatment conditions. **(D)** Representative macroscopic images of MDA-MB-231 xenograft tumors from nude mice. **(E-G)** Dynamic body weight, tumor volume and final tumor weight of mice in each group. **(H)** H&E staining of major visceral organs observed at two magnifications (scale bars: 200 μm, 50 μm). Data are presented as mean ± SEM. ns, not significant. **P*<0.05, ***P*<0.01, ****P*<0.001, *****P*<0.0001 vs. the corresponding control group.

**Supplementary Figure S5: BBR induces PD-L1 degradation through autophagy-lysosome fusion modulated by autophagy initiation and lysosomal formation.**

**(A, B)** Representative immunofluorescence co-localization of PD-L1 and LC3B in MDA-MB-231 and BT-549 cells under different treatments. Relative fluorescence intensity was quantified to reflect changes in PD-L1 and LC3B abundance. **(C, D)** Co-staining of lysosomal marker LAMP1 and PD-L1 in two TNBC cell lines. Quantitative results illustrate the regulatory effects of BBR, BafA1 and 3-MA on LAMP1 expression and PD-L1 stability. **(E, F)** Double immunofluorescence staining of LC3B and LAMP1. Quantitative analysis reveals the impacts of different treatments on autophagosome-lysosome fusion process. Scale bar = 20 μm (n=3 independent experiments for each condition). Data are presented as mean ± SEM. ns, not significant. **P*<0.05, ***P*<0.01, ****P*<0.001, *****P*<0.0001 vs. the corresponding control group.
