# Supplementary material for: Berberine induces PD-L1 degradation via the autophagy-lysosome pathway through the PI3K-Akt pathway and enhances immunogenic cell death in triple-negative breast cancer
Source: Front Immunol. 2026 Aug 7;17:1797840. doi: 10.3389/fimmu.2026.1797840 (PMC13493310; doi:10.3389/fimmu.2026.1797840)
Supplement: Supplementary file 2 [file SupplementaryFile1.docx]

**Berberine induces PD-L1 degradation via the autophagy-lysosome pathway through the PI3K-Akt pathway and enhances immunogenic cell death in triple-negative breast cancer**

Yuqin Wei^1^, Qiudan Huang^2^, Xingwen Wei^3^,Ruilan Mo^2^, Wei Zhao^1*^

^1^ Department of Comprehensive Cancer Center (Ward 2), The Second Affiliated Hospital of Guangxi Medical University, Nanning, China

^2^ Department of Radiotherapy, Guangxi Medical University Cancer Hospital, Nanning, China

^3^ Department of Respiratory Medicine, Wuming Hospital of Guangxi Medical University, Nanning, China

* Corresponding author: Department of Comprehensive Cancer Center (Ward 2), The Second Affiliated Hospital of Guangxi Medical University, Nanning, China. E-mail address: zhaowei@gxmu.edu.cn.

**Supplementary Tables**

**Supplementary Table S1. Sources of key experimental reagents used in this study**

| Reagent Name | Vendor/Catalog Number |
| --- | --- |
| Bafilomycin A1 (BafA1) | Ambeed, A614767 |
| Proteasome inhibitor (MG132) | Ambeed, A541748 |
| 3-Methyladenine (3-MA) | MCE, HY-19312 |
| Chloroquine (CQ) | Beyotime, Y264393 |
| Cycloheximide (CHX) | GLPBIO, GC17198 |
| Recombinant human interferon-γ (IFN-γ) | PeproTech, AF-300-02-20 |
| Tribromoethanol (1.25%, anesthetic) | meilunbio, MA0478 |
| Human PD-L1 ELISA Kit | Jianglai Bio, JL19324 |
| Cytotoxicity LDH Assay Kit | Nanjing Jiancheng, A020-2-2 |
| Membrane Protein Extraction Kit | Solarbio, EX1110 |
| Primary antibody: PD-L1 | Abcam, ab205921 |
| Primary antibody: Na+/K+-ATPase | ABclonal, A11683 |
| Primary antibody: Calreticulin (CRT) | ABclonal, A1066 |
| Primary antibody: HMGB1 | Solarbio, K106630P |
| Primary antibody: HSP70 | ABclonal, A12948 |
| Primary antibody: LC3B | Cell Signaling Technology, 83506S |
| Primary antibody: P62 | Abways, CY5546 |
| Primary antibody: PD-1 [Alexa Fluor® 488] | Bio-techne, NBP3-28113AF488 |
| Primary antibody: CD3 | BioLegend, 317301 |
| Primary antibody: CD28 | BioLegend, 302901 |
| IL-2 | Cloud-clone, RPA073Hu01 |
| Lyso-Tracker Red (lysosome-specific probe) | Beyotime, C1046 |
| Acridine Orange (AO, lysosomal membrane stability probe) | Ambeed, A283980 |
| Calcein-AM (live cell labeling probe) | Beyotime, C2012 |
| Hoechst 33342 (nuclear stain) | Solarbio, DA0013 |
| Total RNA Extraction Kit (Cells/Tissues) | New Saimei, M5106 |
| Enhanced BCA Protein Assay Kit | Beyotime, P0010S |
| qRT Master Mix (+gDNA Remove) | Yungen Bio, qRT001 |

**Supplementary Table S2: Clinical Characteristics of Human Samples**

| Sample Type | Sample ID | Age (y) | Tumor Subtype | Clinical Stage (AJCC 8th) | Pathological Grade | Pre-treatment Collection |
| --- | --- | --- | --- | --- | --- | --- |
| Tissue | AAA | 42 | TNBC | T1cN0M0, IA | Invasive ductal carcinoma, Grade 3 | Yes |
| Tissue | BBB | 36 | TNBC | ypTisN0M0, 0 | Invasive ductal carcinoma, Grade 3 | Yes |
| Tissue | CCC | 46 | Luminal B1 | pT2N1M0, IIB | Invasive ductal carcinoma, Grade 2 | Yes |
| Serum (Cancer) | DDD | 35 | Luminal B1 | pT1cN3M0, IIIC | Invasive ductal carcinoma | Yes |
| Serum (Cancer) | EEE | 34 | Luminal B1 | cT3N1M0, IIIA | Invasive ductal carcinoma, Grade 2 | Yes |
| Serum (Cancer) | FFF | 43 | Luminal B1 | cT4N0M0, IIIB | Invasive ductal carcinoma, Grade 2 | Yes |
| Serum (Cancer) | GGG | 53 | Luminal B1 | pT2N1M0, IIB | Invasive ductal carcinoma, Grade 2 | Yes |
| Serum (Healthy) | N1-N4 | 20-  45 | - | - | - | - |

Notes: Inclusion/Exclusion Criteria for Human Sample Collection

Inclusion Criteria:

Pathologically confirmed primary breast cancer patients (for cancer samples) and healthy volunteers with no history of malignant tumors (for healthy serum samples);

Samples collected at initial diagnosis and without any prior anti-tumor treatment (surgery, chemotherapy, radiotherapy, targeted/immunotherapy/endocrine therapy);

Complete clinical and pathological data (tumor subtype, stage, grade, etc.) available;

The participants have been verbally informed.

Exclusion Criteria:

Patients with recurrent or metastatic breast cancer;

Patients with other malignant tumors or severe systemic diseases (cardiovascular, liver, kidney, etc.);

Patients who received anti-tumor treatment before sample collection;

Sample quality that does not meet experimental requirements (e.g., hemolysis of serum samples, severe necrosis of tissue samples).

**Supplementary Table S3: Antibodies for flow cytometry**

| Target Marker | Fluorochrome | Clone | Vendor | Catalog No. | Working Concentration | Staining Type |
| --- | --- | --- | --- | --- | --- | --- |
| CD3ε | APC | 500A2 | Biolegend | 152305 | 0.25 μg/100 μL cells | Surface |
| CD8a | PerCP/Cyanine5.5 | 53-6.7 | Biolegend | 100733 | 1 μg/100 μL cells | Surface |
| CD25 | PE/Cyanine7 | 3C7 | Biolegend | 101915 | 0.5 μg/100 μL cells | Surface |
| CD69 | APC/Cyanine7 | H12F3 | Biolegend | 126403 | 0.25 μg/100 μL cells | Surface |
| FOXP3 | PE | MF-14 | Biolegend | 126403 | 1 μg/100 μL cells | Intracellular (transcription factor) |
| Granzyme B | Brilliant Violet 421™ | QA18A28 | Biolegend | 396413 | 5 μL/100 μL cells | Intracellular |
| PD-L1 | PE | MIH1 | BD Biosciences | 557924 | 0.5 μg/100 μL cells | Surface |
| CD44 | PE | IM7 | Selleck | G0077 | 0.5 μg/100 μL cells | Surface |
| PE-conjugated mouse IgG2b isotype control | PE | 27-35 | BD Biosciences | 556656 | 0.25 μg/100 μL cells | Surface (isotype control) |

**Supplementary Methods**

**S1. Lyso-Tracker Red staining**

Lyso-Tracker Red is an acidophilic fluorescent dye whose fluorescence intensity increases under acidic conditions and is positively correlated with the number and acidity of lysosomes. After cell treatment, 50 nmol/L Lyso-Tracker Red was added and incubated at 37°C for 30 min. After washing with PBS, the red fluorescence intensity of lysosomes (reflecting lysosomal activity and acidification degree) was observed under a fluorescence microscope.

**S2. Acridine orange staining**

Normal lysosomes exhibit red fluorescence, while damaged lysosomal membranes show green fluorescence; a decreased red-green ratio indicates reduced lysosomal membrane stability. After cell treatment, 1 μg/mL acridine orange was added and incubated at 37°C for 15 min. After washing with PBS, the cells were observed under a fluorescence microscope, and the red-green fluorescence ratio (reflecting lysosomal membrane stability) was quantified.

**S3. Detailed procedure for PBMC isolation and activation**

Peripheral blood mononuclear cells (PBMCs) were isolated from fresh whole blood of healthy donors (Ethics approval No. WM‑2026(22)) by the Human Peripheral Blood Mononuclear Cell Separation Kit (Solaibio, P8680). Briefly, 5 mL of fresh blood was diluted with an equal volume of diluent. A density gradient was prepared by layering Reagent A (3 mL) followed by Reagent D (2 mL) in a 15 mL sterile tube. The diluted blood was carefully layered onto the gradient and centrifuged at 800 × g for 30 min at room temperature using a horizontal rotor. The white mononuclear cell layer was collected, washed twice with washing solution, and resuspended in RPMI-1640 with 10% FBS.

Lymphocytes were enriched from PBMCs using the differential adherence method as recommended by the kit manufacturer’s protocol. Briefly, PBMCs were resuspended in mononuclear cell culture medium at a density of 1×10^6^ cells/mL and seeded into cell culture plates. Monocytes, which exhibit rapid adherence properties, were allowed to adhere during a 4 h incubation at 37°C in a 5% CO_2_ humidified incubator. After incubation, the non-adherent cell fraction—enriched for lymphocytes—was carefully collected by gentle pipetting to minimize both the loss of non-adherent lymphocytes and the disturbance of the adherent monocyte layer. We acknowledge that this method has inherent limitations, including potential T cell loss due to nonspecific adhesion and residual contamination with non-T mononuclear cells.

For T‑cell activation, 24‑well plates were pre‑coated with 1 μg/mL agonistic anti‑human CD3 antibody in PBS overnight at 4 ℃, washed twice with PBS, and 1 mL of the cell suspension was added per well. Soluble anti‑human CD28 antibody (0.5 μg/mL) and recombinant human IL‑2 (50 U/mL) were supplemented at the time of seeding. Cells were incubated at 37 ℃ with 5% CO_2_. T‑cell clustering, indicative of early activation, was evident at 24 h. For functional assays, cells were activated for 72 h, when extended culture was required, half of the medium was replaced on day 3 with fresh medium containing 50 U/mL IL‑2. After activation, cells were harvested, washed twice with PBS (300 × g, 5 min), and resuspended in fresh medium before co‑culture with target cells.

**S4. LDH release assay**

**S4.1 Experimental groups**

Table S4 Experimental grouping and design of the LDH release assay

| Group | Composition | Experimental Purpose |
| --- | --- | --- |
| NC  (negative control) | Untreated tumor cells + culture medium (no effector lymphocytes) | Measure spontaneous LDH leakage from untreated tumor cells (background) |
| BBR | BBR-pretreated tumor cells + culture medium (no effector lymphocytes) | To assess direct cytotoxicity of BBR alone on tumor cells (previously confirmed by viability assays to show no significant toxicity) |
| Lymphocyte control | Activated T cells + culture medium (no tumor cells) | Detect spontaneous LDH release from effector lymphocyte population (background) |
| NC+Lymphocyte | Untreated tumor cells + activated lymphocyte-enriched PBMCs | Baseline tumor lysis induced by activated lymphocyte population without BBR intervention |
| BBR+Lymphocyte | BBR-pretreated tumor cells + activated lymphocyte-enriched PBMCs | Quantify altered lymphocyte-mediated tumor cytotoxic potency upon BBR tumor cell preconditioning (experimental group) |
| Maximum | Tumor cells + 1% Triton X-100 (no effector cells) | Maximum LDH release (positive control, complete cell lysis) |
| Blank  (medium only) | Pure complete culture medium only | To subtract background absorbance |

**S4.2 Experimental Procedure**

MDA-MB-231 and BT-549 cells (5 × 10^3^ cells/well) were seeded in 96-well plates and treated with 10 μmol/L BBR or vehicle (DMSO) for 24 h. After drug removal and washes, tumor cells were maintained as adherent monolayers without detachment or re-counting to preserve physiological cell–cell interactions and avoid trypsinization-induced surface protein alteration. Lymphocyte-enriched PBMCs, activated with anti-CD3/anti-CD28 antibodies plus IL-2 for 72 h, were added at an E:T ratio of 5:1 (2.5 × 10^4^ cells/well) based on the initial seeding density. Each group was performed in triplicate. After 24 h of co-culture at 37 °C, supernatants were collected and centrifuged at 1,200 × g for 5 min. LDH activity was measured using an LDH assay kit (Nanjing Jiancheng, Cat. No. A020-2-2) according to the manufacturer’s instructions. Complete lysis controls were prepared with 1% Triton X-100. The specific killing rate was calculated as: Absorbance at 440 nm was measured using a microplate reader. The killing rate was calculated as follows: Killing rate (%) = [(OD value of experimental group - OD value of blank group) / (OD value of maximum release group - OD value of blank group)] × 100%.

**S5. Calcein-AM staining assay**

Calcein AM has very low cytotoxicity and minimally affects cellular functions such as cell proliferation or lymphocyte chemotaxis. TNBC cells were seeded in 24-well plates and cultured until adherent. Cells were treated with 10 μmol/L BBR or vehicle DMSO for 24 h. After PBS washing, tumor cells were co-cultured with CD3/CD28 and IL-2-activated lymphocyte-enriched PBMCs at an E:T ratio of 5:1 for 24 h.

For Calcein-AM staining (Beyotime, C2012), a working solution of 1 μmol/L Calcein-AM was prepared according to the manufacturer’s protocol. Cells were incubated with Calcein-AM working solution at 37℃ for 30 min in the dark, followed by incubation in fresh medium for another 30 min. After thorough PBS washing, viable TNBC cells with green fluorescence were observed and imaged under a fluorescence microscope.

For quantitative analysis, cell culture supernatants were collected and transferred to 96-well plates. Fluorescence intensity was detected at an excitation wavelength of 494 nm and an emission wavelength of 514 nm (CLARIOstar Plus, BMG LABTECH) .The cell killing rate was calculated according to the following unified formula:

$$Specific Lysis (\%) = \frac{F_{(Co-culture - Tumor cell alone)}}{F_{(Max release - Tumor cell alone)}}\times100\%$$

For co-culture refers to the fluorescence value of tumor cells co-cultured with activated PBMCs (NCT in the control setting, BBRT in the BBR treatment setting), FTumor cell alone refers to the fluorescence value of tumor cells cultured without PBMCs (NC in the control setting, BBR in the BBR treatment setting), FMax release is the maximal fluorescence value from complete cell lysis induced by 1% Triton X-100.

**S6. CCK-8 assay for evaluating PBMC-mediated cytotoxicity**

MDA-MB-231 and BT-549 cells were seeded in 96-well plates at a density of 6 × 10^3^ cells/well. After adherence, TNBC cells were pretreated with 100 ng/mL IFN-γ for 24 h to simulate the inflammatory immunosuppressive tumor microenvironment, followed by 10 μmol/L BBR treatment for another 24 h. Subsequently, activated lymphocyte-enriched PBMCs were added at a tumor cell:immune cell ratio of 1:5 for 24 h co-culture. After co-culture, suspended dead cells and debris were removed by PBS washing. CCK-8 working solution was prepared by diluting the stock reagent at a 1:10 ratio with complete medium, and 100 μL of working solution was added to each well. After incubation at 37 °C for 2 h, the absorbance value at 450 nm was measured to calculate relative tumor cell viability. Three biological replicates were set for each group.

**S7. Crystal violet staining assay for viable TNBC cells**

TNBC cells were seeded in 24-well plates and cultured to adherence. Cells were serum-starved for 24 h and pretreated with 100 ng/mL IFN-γ, followed by 10 μmol/L BBR intervention. Activated lymphocyte-enriched PBMCs were added at a tumor cell:immune cell ratio of 1:5 for 24 h co-culture. After incubation, supernatants and non-adherent immune cells were discarded, and plates were gently washed with PBS to remove dead cells. TNBC cells were fixed with 4% paraformaldehyde for 20 min and stained with 0.5% crystal violet solution for 20 min. After PBS washing and natural air-drying, viable TNBC cells were visualized and photographed under a light microscope.

**S8. Analysis of tumor-infiltrating lymphocytes (TILs)**

Fresh 4T1 tumor tissues were harvested and processed into single-cell suspensions using optimized enzyme digestion. Briefly, tumor tissues were minced into small pieces (approximately 1-2 mm^3^) and incubated with digestion buffer containing collagenase type IV (1 mg/mL), hyaluronidase (0.5 mg/mL), and deoxyribonuclease I (DNase I, 0.1 mg/mL) in RPMI-1640 medium at 37°C for 1 hour with gentle agitation. The digested tissue was filtered through a 70 μm cell strainer to obtain a single-cell suspension. Cells were washed twice with PBS and resuspended in flow cytometry staining buffer (PBS containing 2% FBS).

For TIL analysis, 2 × 10^6^ cells per sample were used. First, cells were stained with Zombie Green™ fixable viability dye (1:2000 dilution, BioLegend, catalog number 423102) for 15 min at room temperature in the dark to discriminate live from dead cells. After viability staining, cells were washed twice with PBS and incubated with Fc receptor blocking buffer (anti-mouse CD16/32 antibody, 1:2000 dilution, BioLegend, catalog number 101319) for 10 minutes at 4°C to prevent non-specific antibody binding. Subsequently, cells were stained with surface marker antibodies at 4°C in the dark for 30 min. The following antibodies were used for surface staining: Anti-mouse CD3 conjugated with allophycocyanin (APC) (clone 145-2C11, BD Biosciences, catalog number 100236, 1:100 dilution), Anti-mouse CD8 conjugated with PerCP/Cyanine5.5 (clone 53-6.7, BD Biosciences, catalog number 100732, 1:100 dilution), Anti-mouse CD25 conjugated with PE/Cyanine7 (clone PC61, BD Biosciences, catalog number 560631, 1:100 dilution), and Anti-mouse CD69 conjugated with APC/Cyanine7 (clone H1.2F3, BD Biosciences, catalog number 560703, 1:100 dilution).

After surface staining, cells were washed twice with PBS and fixed and permeabilized using the FoxP3/Transcription Factor Staining Buffer Set (eBioscience, catalog number 00-5523-00) according to the manufacturer’s instructions. Briefly, cells were fixed with fixation/permeabilization buffer for 30 min at 4°C, washed twice with permeabilization buffer, and then stained with intracellular markers at 4°C in the dark for 30 minutes. The following antibodies were used for intracellular staining: Anti-mouse FOXP3 conjugated with PE (clone FJK-16s, eBioscience, catalog number 12-5773-82, 1:100 dilution) and Anti-mouse Granzyme B conjugated with Brilliant Violet 421 (BV421) (clone GB11, BioLegend, catalog number 372220, 1:100 dilution). After intracellular staining, cells were washed twice with permeabilization buffer, resuspended in 300 μL of PBS containing 1% FBS, and analyzed by flow cytometry.

Gating strategy for TIL analysis: A sequential gating strategy was employed to accurately identify and quantify distinct lymphocyte subsets within tumor-infiltrating lymphocyte populations. First, singlet cell populations were gated based on FSC-H vs FSC-A to exclude cell aggregates and doublets. Subsequently, live cells were gated by excluding Zombie Green™-positive dead cells from singlet populations. Third, CD3-positive T cell populations were gated from live cell gates to establish a comprehensive T cell analysis framework. Fourth, CD8-positive cytotoxic T cells were identified from CD3-positive T cells, and this subset was further analyzed for activation status by gating CD69-positive cells; the percentage of CD69-positive cells relative to total CD3-positive CD8-positive T cells was quantified to assess T cell activation status. Concurrently, Granzyme B-positive cytotoxic T cells were gated and quantified as a percentage of CD8-positive T cells to evaluate cytotoxic potential using GzmB fluorescence intensity on the X-axis and FSC-A on the Y-axis. For regulatory T cell analysis, CD4-positive T cells were first identified as the CD8-negative fraction within the CD3-positive population. Within the CD3-positive CD8-negative subset, regulatory T cells were identified as CD25-positive Foxp3-positive double-positive cells and quantified as a percentage of CD4-positive T cells to assess immunosuppressive T cell populations. This hierarchical gating strategy ensured accurate discrimination of T cell subsets and their functional phenotypes, with strict quality control at each step to minimize non-specific background and ensure data reliability.
